# Imipramine and olanzapine block apoE4-catalyzed polymerization of Aβ and show evidence of improving Alzheimer’s disease cognition

**DOI:** 10.1186/s13195-022-01020-9

**Published:** 2022-06-29

**Authors:** Noah R. Johnson, Athena C.-J. Wang, Christina Coughlan, Stefan Sillau, Esteban Lucero, Lisa Viltz, Neil Markham, Cody Allen, A. Ranjitha Dhanasekaran, Heidi J. Chial, Huntington Potter

**Affiliations:** Department of Neurology, University of Colorado Alzheimer’s and Cognition Center, Linda Crnic Institute for Down Syndrome, University of Colorado, Anschutz Medical Campus, Aurora, CO USA

**Keywords:** Amyloid-β, Apolipoprotein E, Dementia, High-throughput screening, Antidepressant, Antipsychotic

## Abstract

**Background:**

The apolipoprotein E (*APOE*) ε4 allele confers the strongest risk for late-onset Alzheimer’s disease (AD) besides age itself, but the mechanisms underlying this risk are debated. One hypothesis supported by evidence from multiple labs is that apoE4 binds to the amyloid-β (Aβ) peptide and catalyzes its polymerization into neurotoxic oligomers and fibrils. Inhibiting this early step in the amyloid cascade may thereby reduce or prevent neurodegeneration and AD.

**Methods:**

Using a design of experiments (DOE) approach, we developed a high-throughput assay to identify inhibitors of apoE4-catalyzed polymerization of Aβ into oligomers and fibrils. We used it to screen the NIH Clinical Collection of small molecule drugs tested previously in human clinical trials. We then evaluated the efficacy and cytotoxicity of the hit compounds in primary neuron models of apoE4-induced Aβ and phosphorylated tau aggregation. Finally, we performed retrospective analyses of the National Alzheimer’s Coordinating Center (NACC) clinical dataset, using Cox regression and Cox proportional hazards models to determine if the use of two FDA-approved hit compounds was associated with better cognitive scores (Mini-Mental State Exam), or improved AD clinical diagnosis, when compared with other medications of the same clinical indication.

**Results:**

Our high-throughput screen identified eight blood-brain barrier (BBB)-permeable hit compounds that reduced apoE4-catalyzed Aβ oligomer and fibril formation in a dose-dependent manner. Five hit compounds were non-toxic toward cultured neurons and also reduced apoE4-promoted Aβ and tau neuropathology in a dose-dependent manner. Three of the five compounds were determined to be specific inhibitors of apoE4, whereas the other two compounds were Aβ or tau aggregation inhibitors. When prescribed to AD patients for their normal clinical indications, two of the apoE4 inhibitors, imipramine and olanzapine, but not other (non-hit) antipsychotic or antidepressant medications, were associated with improvements in cognition and clinical diagnosis, especially among *APOE4* carriers.

**Conclusions:**

The critical test of any proposed AD mechanism is whether it leads to effective treatments. Our high-throughput screen identified two promising FDA-approved drugs, imipramine and olanzapine, which have no structural, functional, or clinical similarities other than their shared ability to inhibit apoE4-catalyzed Aβ polymerization, thus identifying this mechanism as an essential contribution of apoE4 to AD.

**Supplementary Information:**

The online version contains supplementary material available at 10.1186/s13195-022-01020-9.

## Background

Genetic factors can increase the risk for developing AD, in particular in individuals who carry the ε4 allele of the *APOE* gene [1]. The three common apoE isoforms, apoE2, apoE3, and apoE4, differ by single amino acid substitutions at positions 112 and 158. Of the three common allelic variants of *APOE*, ε3 is most prevalent, accounting for 70–80% of the total alleles in the human population, followed by ε4, which accounts for 10–15%, and then ε2, which accounts for 5–10% [2]. Carrying one copy of *APOE4* more than triples the risk for AD, whereas being homozygous for *APOE4* increases the risk by greater than 12-fold [1]. Indeed, despite its low allelic frequency in the general population, approximately 60–65% of individuals with AD carry at least one copy of *APOE4* [3]. The onset of AD symptoms occurs earlier in *APOE4* carriers than in non-carriers and is accompanied by more severe plaque deposition, intraneuronal Aβ accumulation, cerebral amyloid angiopathy, and BBB dysfunction [1, 4, 5].

Multiple potential mechanisms by which apoE4 increases the risk for AD have been proposed and investigated. For example, apoE, and especially apoE4, binds to Aβ with high affinity and acts as a catalyst to accelerate the rate of Aβ oligomer and fibril formation [6–9], increase their stability [10, 11], and promote their neurotoxicity [12–14]. Consistent with this premise, human apoE4 expressed in mice seeded Aβ aggregation [15], and conversely, knockout of the mouse *Apoe* gene in transgenic mice expressing human amyloid precursor protein (APP) abolished amyloid fibril and plaque formation and cognitive decline [16, 17]. Furthermore, careful longitudinal evaluation in prodromal AD has revealed that *APOE* genotype plays the greatest role during the initial seeding stages of Aβ deposition and that *APOE4* genotype is strongly associated with increased Aβ oligomer levels in the brain [18–20]. Additional contributors to the increased genetic risk of *APOE4* in AD may include impaired Aβ clearance, exacerbated oxidative stress and neuroinflammation (reviewed in [21–23]), and loss of critical apoE functions. Notably, apoE is found co-deposited in amyloid plaques in the AD brain, suggesting a direct interaction with Aβ [24]. Rare mutations in the Aβ binding domain of apoE markedly reduce the risk for AD in humans [25, 26]. Taken together, substantial evidence supports a role for apoE as an essential molecular chaperone for Aβ aggregation in the brain and suggests that inhibiting this process is a promising therapeutic approach to preventing AD.

ApoE-targeted therapeutics for AD have focused predominantly on modulating the overall levels of apoE or the degree of its lipidation. ApoE depletion in AD mouse models has been accomplished using antisense oligonucleotides, immunotherapies, or tamoxifen-inducible *APOE* repression, each of which was found to reduce amyloid pathology [27–29]. However, given that apoE is expressed throughout the body where it carries out many critical functions, a reduction in total apoE levels is expected to have many undesirable side effects [30]. Thus, focusing on the interaction between apoE and Aβ may yield a more precise therapeutic benefit for AD without interfering with the many beneficial functions of apoE. Small molecule “structure correctors” or gene editing have been used to block the formation of the pathological conformation of apoE4 [31, 32]. Additionally, synthetic peptides or peptoids designed to block the apoE-binding site on Aβ were also found to reduce Aβ aggregation in vitro and in AD mouse models [12, 33, 34]. Although the clinical translatability of these therapies remains to be determined, together, they validate the inhibition of the apoE4-Aβ interaction as a tractable therapeutic approach for AD.

Here, we describe the identification of a set of small molecule drugs that can block the interaction between apoE4 and Aβ. We developed an apoE4-catalyzed Aβ fibrillization assay and employed it for high-throughput screening (HTS) of the National Institutes of Health (NIH) Clinical Collection (NCC) library of small molecules with a history of use in clinical trials, many of which are Food and Drug Administration (FDA)-approved drugs. Repurposing known drugs has numerous benefits, such as the availability of safety and dosing information that allows for faster and more cost-effective clinical testing. Through a series of HTS assays, we identified eight hit compounds that reduced apoE4-catalyzed Aβ fibrillization in a dose-dependent manner. We present evidence that two of those hit compounds — imipramine and olanzapine — reduced Aβ and phosphorylated tau (pTau) neuropathology in cell culture models and, when taken by AD patients for their other normal clinical indications, were associated with improved cognition and greater incidence of receiving an improved clinical diagnosis. Because imipramine and olanzapine are completely different drugs with regard to their structures, designed mechanisms of action, and current approved clinical indications, and their only common link is our discovery of their shared ability to block the apoE4-catalyzed polymerization of Aβ into neurotoxic fibrils, these findings validate this mechanism as an essential contribution of apoE4 to AD.

## Methods

### Development of an apoE4-Aβ fibrillization assay

Recombinant human Aβ42 sodium hydroxide (NaOH) salt (rPeptide) was received following pre-treatment to ensure a consistent monomeric preparation, as described previously [35]. For NaOH pre-treatment of Aβ peptide, briefly, following recombinant protein expression and purification, Aβ42 peptides were dissolved in 2 mM NaOH, pH 10.5, and then sonicated and lyophilized. Upon receipt, the lyophilized peptide was reconstituted in ice-cold Dulbecco’s phosphate-buffered saline (DPBS), pH 7.4, which avoids the solution passing through the isoelectric point of Aβ (pI = 5.5), which would induce aggregation [36]. The reconstituted Aβ42 stock solution was quickly aliquoted and snap-frozen in liquid nitrogen and then stored at −80°C until use. Great care was taken to ensure consistency and reproducibility across all experiments by using Aβ from a single batch, thawing and maintaining Aβ stocks on ice until use, and never re-freezing the unused portion of thawed Aβ stocks. For fibrillization experiments, Aβ42, recombinant human apoE4 (Sigma), and thioflavin T (ThT; Sigma) were combined at pre-determined concentrations in DPBS in a total volume of 40 μl in a 384-well μ-clear bottomed plate (Greiner). Plates were sealed to prevent evaporation and incubated at 37°C with constant rapid agitation and the fluorescence intensity of ThT at *λ*_ex_= 440 nm and *λ*_em_= 490 nm was measured every 10 min for up to 24 h using a Biotek Synergy HTX fluorescence plate reader and Gen5 v3.11 software (Biotek). Once the optimal concentrations of approximately 20 μM Aβ42, 1 nM apoE, and 15 μM ThT were determined, they were maintained throughout subsequent studies unless noted otherwise. For HTS assay validation, recombinant human Aβ40 (rPeptide), recombinant human scrambled Aβ42 (rPeptide), recombinant human apoE2 and apoE3 (Creative Biomart), recombinant human apolipoprotein A-I (apoA-I; Creative Biomart), human plasma-derived apoE (Sigma), or dimethyl sulfoxide (DMSO; Sigma) were included or substituted at the indicated concentrations. In assay optimization experiments, 3−8 wells were used per group and experiments were replicated one or two times, as indicated in the figure legends. When replicated twice, experiments were performed on different days and in different plates and the results of the two experiments were combined.

### HTS of the NCC library

The NCC library was developed by the National Center for Advancing Translational Sciences (https://ncats.nih.gov/smr). Detailed information about these compounds is available using the NIH Chemical Genomics Center Pharmaceutical Collection browser [37]. The NCC library was received from Evotec, Inc., and contained each compound at 10 mM in DMSO which were aliquoted and stored at −80°C until use. To set up the exploratory drug screen, compounds were thawed, diluted in DMSO, and added at a concentration of 2 μM to Aβ42 (2 μM) in water, followed by the addition of apoE4 (20 nM) and the mixture was incubated at room temperature (rt) for 15 min. The mixture was then divided into three separate wells of a 96-well plate, ThT (8 μM) and glycine (30 mM) were added for a total volume of 125 μL per well, and the plate was incubated at rt for 10 min in the dark. The fluorescence intensity of ThT was then measured using the fluorescence plate reader. The 595 compounds were divided across numerous plates, and compounds on each plate were compared to control wells on the same plate that received Aβ42, apoE4, ThT, and DMSO. Unlike in the exploratory screen, the optimal concentrations of 20 μM Aβ42, 1 nM apoE, and 15 μM ThT were used in the HTS assay because this assay was developed and optimized for 384-well plates after the exploratory screen had already been completed. To set up the HTS assay, compounds were thawed, diluted in DMSO, and added to the Aβ42 in DPBS at final concentrations of 0.25, 2.5, and 25 μM in 5% DMSO/95% DPBS (v/v), followed immediately by the addition of apoE4 and ThT in a total volume of 40 μL per well. Plates were sealed to prevent evaporation and incubated at 37°C with constant shaking and the fluorescence intensity of ThT was measured every 10 min for 24 h using the fluorescence plate reader. The 87 compounds were divided across three separate plates, and compounds on each plate were compared to control wells on the same plate that received Aβ42, apoE4, ThT, and 5% DMSO. The criteria for hit identification were that the compound reduced ThT fluorescence by at least 30% at any concentration and that the effect was generally dose-dependent. For HTS, each compound was tested in 3−4 wells per concentration and experiments were replicated one or two times, as indicated in the figure legends.

### Inhibition of Aβ alone and disaggregation of pre-formed fibrils

Each of the eight hit compounds was added to Aβ42 at 0.25, 2.5, and 25 μM in 5% DMSO/95% DPBS (v/v), followed immediately by the addition of ThT and measurement of fluorescence intensity every 10 min for 24 h. The area under the curve (AUC) of ThT fluorescence intensity was calculated and normalized to control wells receiving Aβ42, ThT, and 5% DMSO. To test compounds for disaggregation of pre-formed Aβ fibrils, Aβ42 and apoE4 were combined and incubated at 37°C for 24 h with constant shaking to induce fibrillization. Pre-formed Aβ fibrils were then divided into separate wells, and compounds were added in a final concentration of 5% DMSO and incubated at rt for 30 min with constant shaking. ThT was added to each well, the plates were incubated at rt for 15 min, and then fluorescence intensity was measured and normalized to control wells receiving only 5% DMSO. To test compounds for disaggregation of pre-formed tau fibrils, 2 μM recombinant human K18 tau peptide (Novus), comprising the microtubule binding domain of the 4R tau isoform, was combined with 2 μM heparin (Sigma) and 300 μM dithiothreitol (Invitrogen) in DPBS and incubated at 37°C for 24 h with constant shaking to induce fibrillization. Pre-formed tau fibrils were then divided into separate wells, and compounds were added in a final concentration of 5% DMSO and incubated at rt for 30 min with constant shaking. ThT (12.5 μM) was added to each well, the plates were incubated at rt for 15 min, and then fluorescence intensity was measured and normalized to control wells receiving only 5% DMSO.

### Transmission electron microscopy (TEM)

Immediately following the measurement of ThT fluorescence intensity, pre-formed Aβ fibrils treated with individual hit compounds, or with DMSO as a control, were applied undiluted to Formvar/carbon-coated copper grids with 300 square mesh (Electron Microscopy Sciences) for 2 min. Grids were gently blotted on filter paper (Whatman) to remove excess fibrils, then washed twice in water and stained with 2% (w/v) uranyl acetate (Electron Microscopy Services) twice for 20 sec each, blotting on filter paper in between each step. Grids were air dried and imaged on a Tecnai G^2^ Spirit BioTwin microscope (FEI) at 80 kV with a side-mount digital camera (AMT Imaging). TEM images were processed and analyzed using Fiji version 2.1.0/1.53c.

### Animals

5xFAD transgenic mice, which express the human *APP* gene harboring the Swedish (K670N/M671L), Florida (I716V), and London (V717I) familial AD mutations, and the human presenilin 1 (*PSEN1*) gene harboring the M146L and L286V familial AD mutations, from two separate transgenes, each driven by the murine Thy1 promoter, were originally developed on a mixed B6/SJL background [38]. 5xFAD mice that had been backcrossed to a congenic C57BL/6J background (Jackson Labs # 034848-JAX) were received and maintained as a hemizygous line by breeding with C57BL/6J mice. TgF344-AD transgenic rats, which express the human *APP* gene harboring the Swedish mutation (K670N/M671L), and the human *PSEN1* gene with the Δ exon 9 mutation, both driven by the mouse prion protein promoter [39], were maintained on a Fischer 344 background. Mice and rats were treated in accordance with the *Guide for the Care and Use of Laboratory Animals*. All procedures were approved by the Institutional Animal Care and Use Committee of the University of Colorado (Animal Welfare Assurance # D16-00171).

### 5xFAD mouse primary neuron cell model

5xFAD mouse pups at postnatal day 1–2 were genotyped using primer probes and real-time polymerase chain reaction analysis of 1-mm tail snip samples. Brains from the mouse pups were then rapidly removed, cerebral cortices were isolated using a sterile razor blade, and tissue samples from multiple pups were pooled for experiments. Primary cultures of neurons were prepared using the Papain Dissociation System (Worthington) according to the manufacturer’s instructions. To prepare neuronal cultures, cortical tissue was dissociated in 20 U/ml papain under constant agitation at 37°C for 45 min. A single cell suspension was obtained by trituration, then papain was inactivated using ovomucoid protease inhibitor and cells were filtered through a 100 μm cell strainer and diluted in warm Neurobasal medium supplemented with Glutamax, B27 supplement, and penicillin/streptomycin (all from Gibco). Cells were seeded at 30,000 cells/cm^2^ in 96-well μ-clear bottomed plates (Ibidi) pre-coated with 10 μg/ml poly-D-lysine (Sigma). Neural cultures were maintained at 37°C in a humidified 5% CO_2_ chamber for 3 days, and then half of the culture medium was replaced with a fresh medium also containing CultureOne supplement (Thermo Fisher), which reduces glial cell proliferation to favor neuronal culture. After 7 days in culture, half of the culture medium was replaced, and 100 nM Aβ42 and 1 nM apoE4 were added, followed by the addition of test compounds at 0.01, 0.1, or 1 μM in a final concentration of 0.5% (v/v) DMSO. Every 3 days following exposure to Aβ and apoE4 and treatment with compounds, half of the culture medium was replaced and the Aβ42, apoE4, and test compounds in DMSO were added to maintain the initial concentrations. At 9 days post-exposure (dpe), wells were fixed for immunocytochemistry and the conditioned media was collected for enzyme-linked immunosorbent assay (ELISA) analysis. The minimum number of mice were used to obtain sufficient numbers of cells to test all compounds in three wells per concentration. Cells from individual mice were pooled and used for all groups to remove the effect of biological variation and to allow us to use fewer mice.

### Immunocytochemistry

At the pre-determined end points, the culture medium was removed, and the cells were washed once with DPBS, fixed in 4% (w/v) paraformaldehyde for 30 min, washed four times with DPBS, and stored at 4°C. The cells were permeabilized with 0.1% Triton X-100 in DPBS for 10 min and then blocked with 3% bovine serum albumin (BSA) in DPBS for 90 min and then incubated overnight at 4°C with primary antibodies in 3% BSA in DPBS. 5xFAD mouse cells were labeled with chicken anti-tau (PhosphoSolutions #1998-TAU, 1:1000) and mouse anti-Aβ (82E1, IBL #10323, 1:500) antibodies. TgF344-AD rat cells were labeled with chicken anti-tau, rabbit anti-Aβ (OC, Millipore #AB2286, 1:500), and mouse anti-pTau (AT8, Sigma #MN1020, 1:250) antibodies. Cells were washed and then incubated with Alexa Fluor Plus-conjugated secondary antibodies (Thermo Fisher, 1:500) for 45 min at rt in 3% BSA in DPBS. Cells were washed, and then nuclei were stained with 1 μg/ml Hoechst 33342 (Thermo Fisher) in DPBS for 10 min. The cells were then washed and imaged on an Olympus IX83 inverted fluorescence microscope. Images of entire wells were captured at 20× magnification and then analyzed using Cell Sens v1.12 software (Olympus).

### Aβ ELISA analysis of conditioned media

Aβ concentration in conditioned medium from individual wells was measured using the human Aβ42 ELISA kit (Thermo Fisher), following the manufacturer’s instructions. Two technical replicates were performed in the ELISA assay for each of three different wells per compound per concentration.

### TgF344-AD rat primary neuron cell model

Brains from TgF344-AD transgenic rat pups at postnatal day 1 were removed, and cortices were isolated using a sterile razor blade. Primary cultures of neurons were prepared from cerebral cortices using the Papain Dissociation System (Worthington) according to the manufacturer’s instructions and were plated and cultured as described above for 5xFAD mouse neurons. Cells from individual rat pups were not pooled but rather were cultured in separate wells. After 7 days in culture, half of the culture medium was replaced, and 100 nM Aβ42 and 1 nM apoE4 were added, followed by the addition of test compounds at 1 μM in a final concentration of 0.5% (v/v) DMSO. Every 3 days thereafter, half of the culture medium was replaced and Aβ42, apoE4, and the test compounds in DMSO were added to maintain the initial concentrations. At 14 dpe, the cells were fixed for immunocytochemistry. The minimum number of rats were used to obtain sufficient numbers of cells to test all compounds in three wells per concentration. Cells from individual rat pups were not pooled in order to evaluate the drug effects on different biological replicates, although each drug and controls were tested on cells derived from the same rats.

### NACC data analysis

The NACC uniform dataset v3 [40] was received on April 17, 2020, and contained standardized longitudinal clinical data on 42,661 subjects seen at Alzheimer’s Disease Research Centers (ADRCs) beginning in September 2005 thru the March 2020 data freeze. Subjects who had reported taking at least one of the eight hit compounds were identified by searching the “DRUGS” column. Only subjects who had at least two clinic visits and reported taking a medication prior to their final clinic visit were considered. Control groups of subjects taking antidepressant medications or antipsychotic medications were identified using the “NACCADEP” or “NACCAPSY” columns, respectively, with subjects who reported only taking imipramine or olanzapine being removed. The groups partially overlapped, as, for example, a subject may have reported using imipramine or olanzapine and then reported using a different antidepressant or antipsychotic medication.

In developing the models, medication was treated as a time-varying explanatory variable in order to accurately model exposure, as subjects’ medication statuses changed over time. When a medication was listed at a given time point, the exposure was assumed to have been started at the mid-point between the current and previous time points and to have lasted until the mid-point between the current and subsequent time points. The mean change in Mini-Mental State Exam (MMSE) score over time was modeled using time slopes, with time-varying drug and covariate interactions as slope modifiers. Longitudinal regression models were developed using a random time slope by subject and a continuous 1st order auto-regressive covariance structure for errors on the same subjects and were fit using MMSE scores extracted from the “NACCMMSE” column, and using subjects’ age and sex, identified in the “NACCAGE” and “SEX” columns, as covariates. Only two-way interactions were considered, and linear effects were assumed. Central limit theorems protect against non-severe departures from normality, and MMSE is a validated scale. *APOE* models were also developed using the presence or absence of an *APOE4* allele as a modifier of the drug effect. The “NACCNE4S” column was evaluated and subjects with a “1” or a “2” were designated *APOE4* carriers, subjects with a “0” were designated *APOE4* non-carriers, and subjects with a “9” (missing data) were excluded. Models were also developed where the baseline MMSE score, recorded at a subject’s first clinic visit, was included as a covariate. Linear combinations of parameters were tested with *T* and *F* tests, and the Satterthwaite method was used to calculate the denominator degrees of freedom. Model outputs were used in power analysis calculations performed in PASS 13 software (NCSS). All tests were two-sided, and 95% confidence intervals were presented for all univariate contrasts.

For reversion and conversion models, Cox proportional hazards models were developed, stratified by clinical diagnosis. The Cox model makes no parametric assumptions about the shape of the underlying hazard function, and stratification permits different underlying hazard functions for different clinical diagnoses. Tests for violation of the proportional hazards assumptions are not available for models with time-varying covariates. Clinical diagnoses were extracted from the “NACCUDSD” column, in which a “1” was considered “normal cognition (NC)”, “2” [cognitively impaired, but not meeting the classical definition for mild cognitive impairment (MCI)] or “3” were considered “MCI”, and “4” was considered “AD”. A “4” in the “NACCUDSD” column indicates a diagnosis of dementia, which may include AD, Lewy body dementia, frontotemporal dementia, etc. However, the “NACCALZD” column indicated that the vast majority of subjects receiving a dementia diagnosis were deemed to be of AD etiology (e.g., 29/32 subjects who took imipramine), and thus herein, we refer to this group collectively as AD patients. Drug exposure was modeled using time-varying covariates and cumulative exposure, controlled for time since last exposure, was selected for antidepressants, while on/off status was selected for antipsychotics. The variance calculations accounted for repeated measures, as the subjects could have multiple reversion/conversion events. Multiple reversion/conversion events were handled by aggregating observations within each subject and then the robust sandwich method was used for standard errors and tests. Subjects with an initial clinical diagnosis of AD were excluded from the risk set for conversion, and subjects with an initial clinical diagnosis of NC were excluded from the risk set for reversion, as they were ineligible for the event. Age and sex were controlled for, and in the interaction models, all two-way interactions between drug exposure, age, and sex were considered.

For the MMSE models, effects were assumed to be linear and only two-way interactions were considered. Linear combinations of parameters were tested with *Z* and *χ*^2^ tests. Hazard was modeled on a logarithmic scale and then the results were transformed back to hazard ratios. All tests were two-sided, and 95% confidence intervals were presented for univariate contrasts. For the mixed medications models, subjects taking doxepin, citalopram, fluoxetine, aripiprazole, or quetiapine were identified by searching the “DRUGS” column, and reversion models were developed as described above. Multiple testing adjustment was not applied because of the exploratory nature of the study and the complexity of the models; however, the conclusions drawn from these models would remain unchanged.

### Statistical analyses

DOE and statistical analyses for the development of the fibrillization assay were performed using Minitab 18. Linear regression and one-way analysis of variance (ANOVA) were performed using GraphPad Prism 8. Following ANOVA, comparisons between multiple groups were done by post hoc testing using the Holm-Šidák method and a *P* < 0.05 was considered statistically significant. All statistical tests were two-sided. Sample sizes, experimental replication, and exact statistical tests used are detailed in the figure legends. Except in the case of the kinetic Aβ fibrillization plots, where measurements were taken repetitively from the same wells, all measurements were taken from distinct samples.

## Results

### Development of an apoE4-catalyzed Aβ fibrillization assay for HTS

**Fig. 1:**
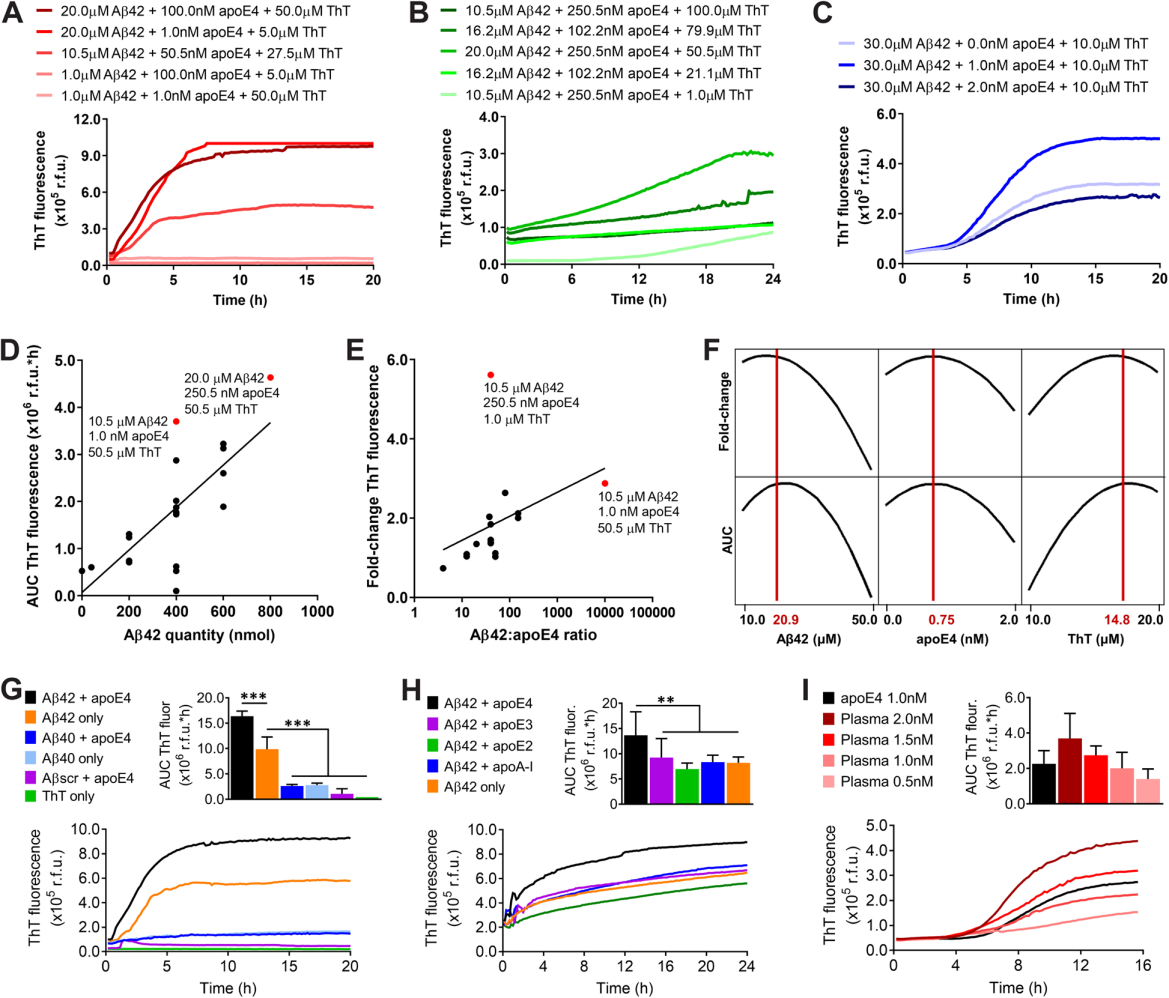
Development of an apoE4-Aβ fibrillization assay for HTS. **a** Concentrations of Aβ42, apoE4, and ThT were varied in a ½ fraction factorial experiment in a 96-well plate and ThT was measured in relative fluorescence units (r.f.u.) in *n* = 3 wells per group. **b** Concentrations of Aβ42, apoE4, and ThT were varied in a response surface experiment in a 384-well plate in *n* = 3−4 wells per group. **c** Concentrations of Aβ42, apoE4, and ThT were varied in a second response surface experiment in a 384-well plate in *n* = 6 wells per group. The experiment was replicated twice, and the results were combined. **a**–**c** Several representative groups were plotted, and the complete results are provided in Additional file 6. **d** The quantity of Aβ42 was plotted against the AUC of ThT fluorescence in r.f.u.*hours (h). Linear regression was performed to identify a best-fit line (*R*^2^ = 0.53). Two assay conditions that maximized the AUC were identified (red dots). **e** The Aβ42 to apoE4 ratio was plotted on a log scale against the fold-change in ThT fluorescence. Linear regression was performed to identify a best-fit line (*R*^2^ = 0.14). Two assay conditions that maximized the fold-change were identified (red dots). **f** In the second response surface experiment, the optimal concentrations of Aβ42, apoE4, and ThT that maximize both the AUC (r.f.u.*h) and the fold-change in ThT fluorescence were identified (red lines). **g** The specificity of the optimized apoE4-Aβ fibrillization assay was evaluated for Aβ42, Aβ40, and a scrambled Aβ42 peptide (Aβ_scr_), with and without apoE4, or for ThT only. The data represent the mean ± SD of *n* = 3 wells per group. ***P < 0.001 by one-way ANOVA. **h** The effect of each human apoE isoform, or of apoA-I, at 1 nM concentration on Aβ42 fibrillization was evaluated. The experiment was replicated twice, and the results were combined. The data represent the mean ± SD of *n* = 7 wells per group. **P < 0.01 by one-way ANOVA. **i** The effect of apoE isolated from human plasma at different concentrations on Aβ42 fibrillization was compared with that of recombinant apoE4. The data represent the mean ± SD of *n* = 4 wells per group

Building on earlier work [6, 7, 10, 12], we adapted an Aβ fibrillization assay monitored with the amyloid-binding dye ThT to study the catalytic effects of apoE4 and optimized it for HTS to identify inhibitors of the apoE4-Aβ interaction. Utilizing a design of experiments (DOE) approach, we first determined the optimal concentrations of Aβ42, apoE4, and ThT to generate a dose-responsive readout. In an initial experiment, we found that lowering Aβ concentration resulted in reduced Aβ fibrillization rate and growth phase duration (Fig. 1a and Additional file 1). We confirmed this result in a second experiment and also found that the baseline level of ThT fluorescence could be reduced by decreasing the concentration of ThT; however, a higher concentration was necessary to observe the maximal ThT fluorescence readout (Fig. 1b and Additional file 2). We also observed that 1 nM apoE4 resulted in greater Aβ fibrillization than did higher concentrations of apoE4, although the effect could be overcome by increasing the concentration of Aβ (Additional file 3), suggesting that the Aβ to apoE4 ratio was important. In a third experiment, we found that 1 nM apoE4 did indeed accelerate Aβ fibrillization, but that increasing apoE4 to 2 nM negated its catalytic effect (Fig. 1c). Consistent with these findings, the integrated AUC of ThT fluorescence increased when a greater quantity of Aβ was used (Fig. 1d), while the fold-change in ThT fluorescence increased with a greater Aβ to apoE ratio (Fig. 1e). Finally, a response optimization algorithm was used to identify the concentrations that maximized both the AUC and the fold-change of ThT fluorescence simultaneously, which was determined to be 20.9 μM Aβ42, 0.75 nM apoE4, and 14.8 μM ThT (Fig. 1f and Additional file 4).

We next evaluated the specificity of our optimized Aβ fibrillization assay. We found that replacing Aβ42 with Aβ40, which is two amino acids shorter, or using a scrambled sequence consisting of the same 42 amino acids, each resulted in significantly less fibrillization (Fig. 1g), consistent with prior reports [41]. We also tested the effect of other apolipoproteins and found that only apoE4 catalyzed Aβ42 fibrillization, while apoE3, apoE2, and apolipoprotein A-I did not (Fig. 1h). To confirm that our results with recombinant human apoE4 were translatable to a normal human population, we then tested apoE isolated from pooled human plasma that contained a mixture of all three apoE isoforms. We found that human plasma-derived apoE catalyzed Aβ42 fibrillization in a dose-dependent manner and at a similar level to that of recombinant apoE4 (Fig. 1i). Finally, to verify the usefulness of our assay for HTS of drug libraries, we added different concentrations of DMSO and found no significant effect up to 10% (v/v) (Additional file 5).

### HTS identifies small molecule inhibitors of apoE4-catalyzed Aβ fibrillization

**Fig. 2:**
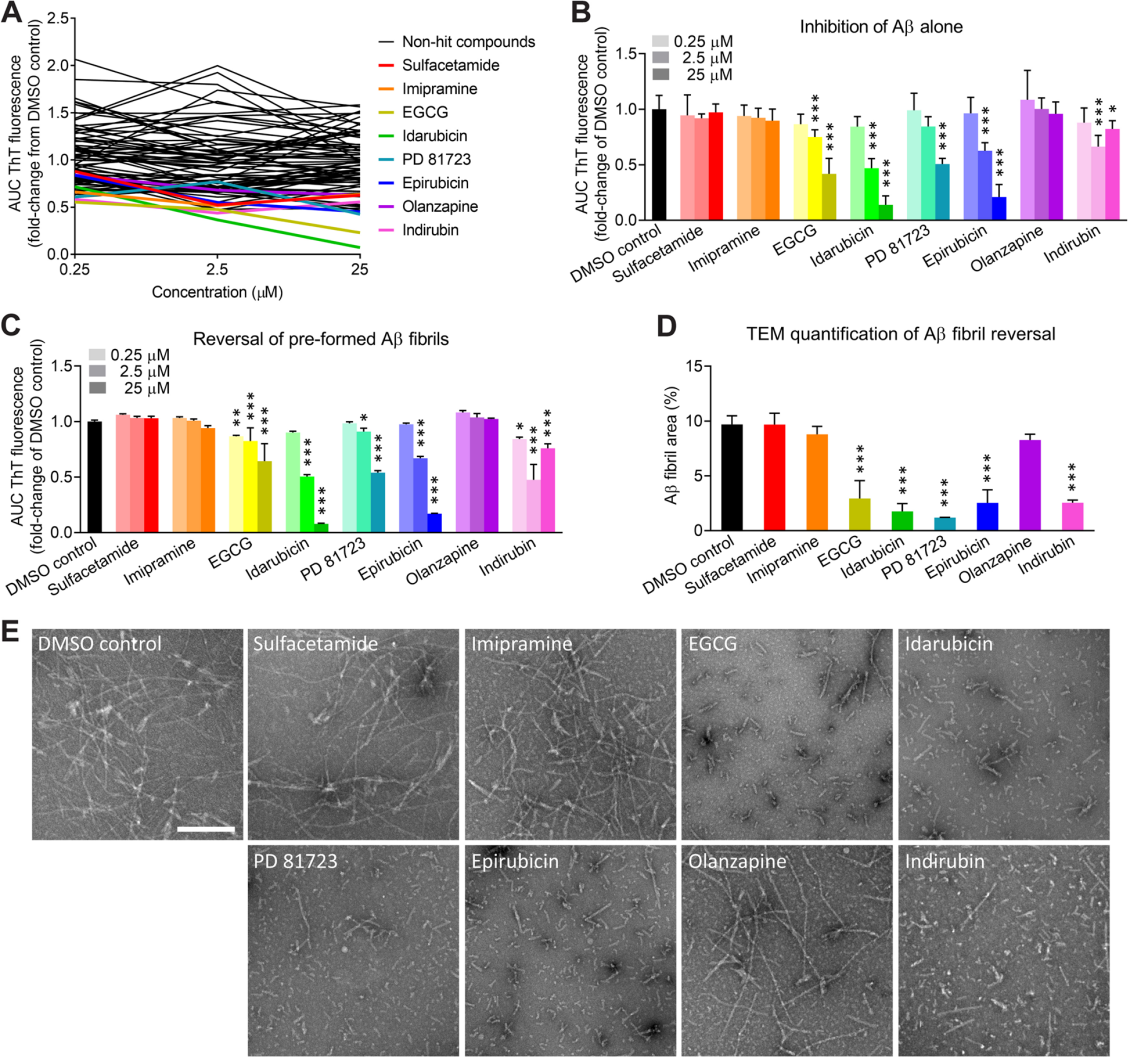
Identification of small molecule compounds that inhibit the apoE4-Aβ interaction or reverse Aβ42 fibril formation. **a** Dose-response experiments with 87 compounds identified in the exploratory screen that were BBB-permeable. Small molecule compounds were added at 0.25, 2.5, and 25 μM in a final concentration of 5% (v/v) DMSO. Eight hit compounds (colored lines) were identified (i.e., sulfacetamide, imipramine, EGCG, idarubicin, PD 81723, epirubicin, olanzapine, and indirubin). The data represent the mean of *n* = 3 wells per concentration for each compound, relative to the mean of *n* = 8 wells for the DMSO control. **b** Eight hit compounds were tested for inhibition of Aβ42 fibrillization independent of apoE4. **c** Eight hit compounds were tested for disaggregation of pre-formed apoE4-catalyzed Aβ42 fibrils. **b**, **c** The experiment was replicated twice, and the results were combined. The data represent the mean ± SD of *n* = 8 wells per concentration for each compound. *P < 0.05, **P < 0.01, and ***P < 0.001 compared to the DMSO control by one-way ANOVA. **d** ApoE4-catalyzed Aβ42 fibrils were treated for 30 min with DMSO or with each compound at 25 μM, except for indirubin, which was tested at 2.5 μM because it had the greatest effect in previous experiments. TEM images were acquired and analyzed for the Aβ fibril area (%). The data represent the mean ± SD of *n* = 3 separate TEM images for each compound. ***P < 0.001 compared to the DMSO control by one-way ANOVA. **e** Representative TEM images of apoE4-catalyzed Aβ42 fibrils treated with DMSO or with each hit compound. Aβ fibrils are observable by negative-stain TEM as light objects on a dark background. Scale bar = 200 nm

The NCC drug library contains small molecule compounds that have a history of use in human clinical trials. We performed an exploratory drug screen of 595 compounds from the NCC library, testing each compound at a concentration of 2 μM, and we identified 134 hits (Additional file 7). We then performed a literature search to determine whether the hit compounds or their metabolites had been reported to be capable of crossing the BBB. Of the 134 hits, we found credible reports that 87 of the compounds had good BBB permeability (Additional file 8). We next analyzed the dose-response effects of these 87 compounds on the kinetics of apoE4-catalyzed Aβ fibrillization in our optimized HTS assay. We identified eight hit compounds (i.e., sulfacetamide, imipramine, epigallocatechin gallate [EGCG], idarubicin, PD 81723, epirubicin, olanzapine, and indirubin) using the criteria that they reduced apoE4-catalyzed Aβ fibrillization by at least 30% and generally displayed a dose-dependent effect (Fig. 2a). The eight hit compounds ranged in size from 214 to 580 Da and had varied chemical structures, although every compound contained at least one aromatic ring (Table 1). One hit, EGCG, was previously shown to inhibit Aβ aggregation in rodent models of AD [42] and is currently being tested in human clinical trials for AD (e.g., NCT03978052), thus validating our overall screening approach.

**Table 1:**
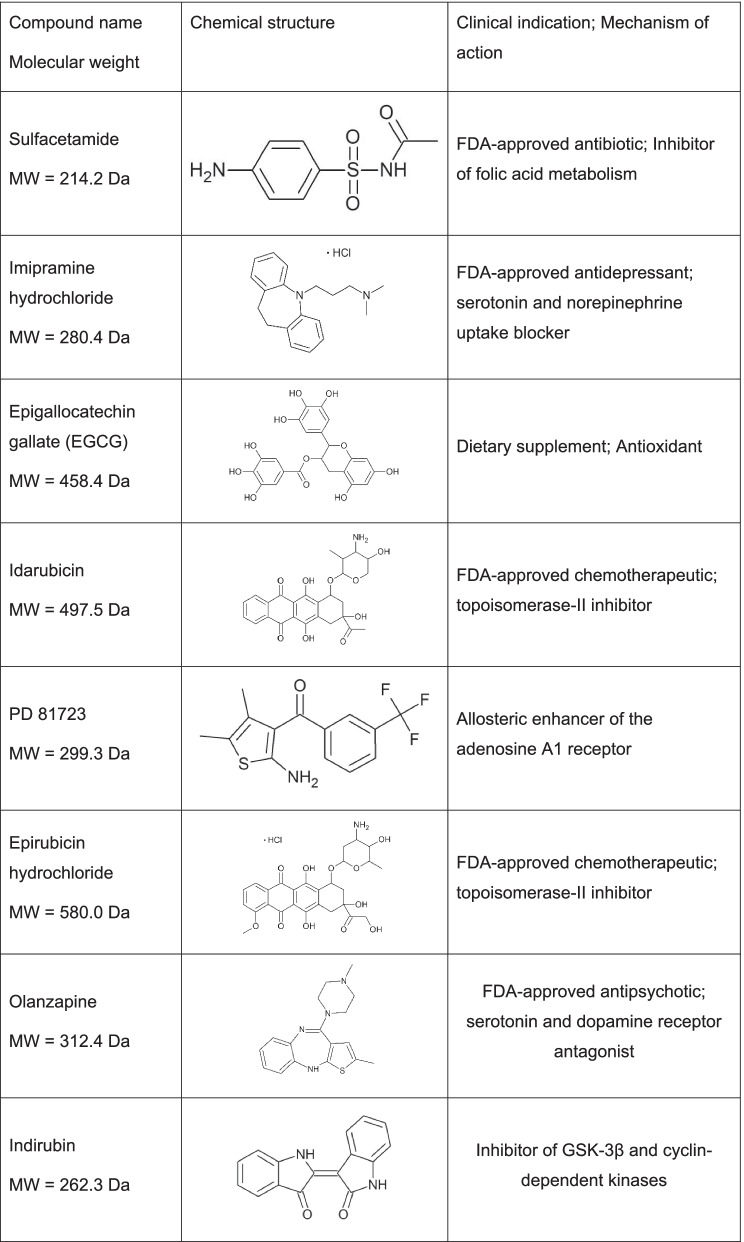
Small molecule inhibitors of apoE4-catalyzed Aβ42 fibrillization

Chemical name, molecular weight, chemical structure, clinical indication (if known), and mechanism of action (if known) of each of the eight hit compounds identified by HTS of the NCC drug library

We sought to identify which compounds were blocking the apoE4-Aβ interaction and which compounds were acting directly on Aβ. EGCG, idarubicin, PD 81723, epirubicin, and indirubin inhibited the fibrillization of Aβ42 alone, independent of apoE4 and in a largely dose-dependent manner (Fig. 2b), suggesting that these five compounds act directly on Aβ. In contrast, sulfacetamide, imipramine, and olanzapine had no effect on the fibrillization of Aβ alone, suggesting that these three compounds are specific inhibitors of the apoE4-Aβ interaction. We also tested the ability of all eight compounds to reverse Aβ fibrillization by first pre-forming apoE4-catalyzed Aβ fibrils and then treating them with each compound. We found that only the five compounds that acted directly on Aβ (i.e., EGCG, idarubicin, PD 81723, epirubicin, and indirubin) could reverse Aβ fibrillization (Fig. 2c). Finally, we used TEM to confirm that these compounds disaggregated Aβ fibrils (Fig. 2d), rather than preventing ThT binding or fluorescence. In contrast to the numerous long Aβ fibrils present following treatment with DMSO, we observed much shorter and fewer Aβ fibrils and aggregates following treatment with the reversal compounds (Fig. 2e). These molecules may be pursued as interventional treatments for patients with pre-existing AD neuropathology.

### Small molecule compounds reduce Aβ neuropathology in primary neurons from 5xFAD transgenic mice

**Fig. 3:**
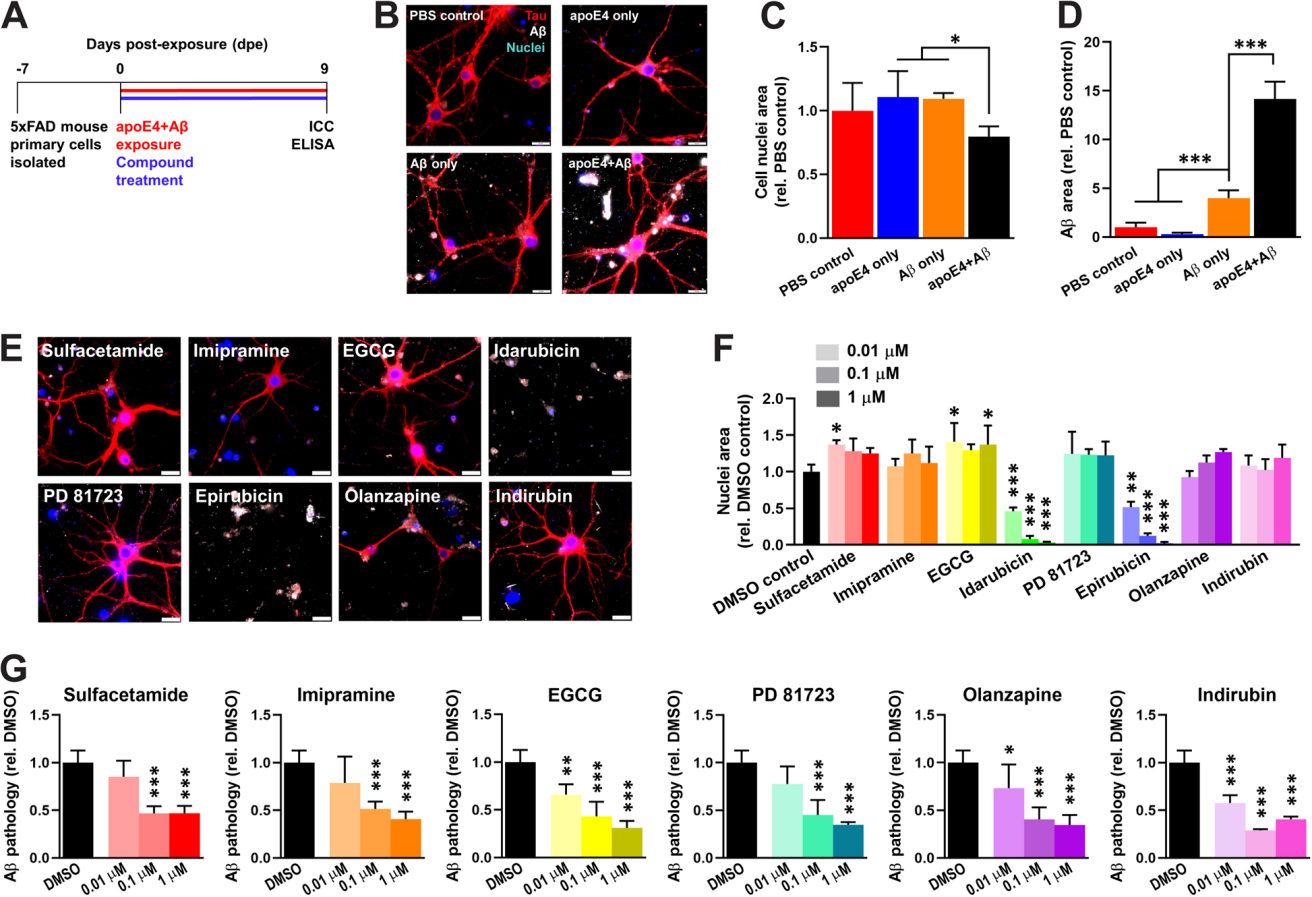
Small molecule compounds inhibit apoE4-catalyzed Aβ pathology in primary neurons from 5xFAD transgenic mice. **a** Schematic of drug efficacy experiments using primary neurons from the 5xFAD transgenic mouse model of Alzheimer’s disease. One week after cell isolation from day P1-P2 pups, cells were exposed to 100 nM Aβ42 and 1 nM apoE4 and were treated concurrently with 0.01, 0.1, or 1.0 μM compound in a final concentration of 0.5% (v/v) DMSO. The cell medium was changed every 3 days by removing half and replacing it with a fresh medium containing Aβ42, apoE4, and compound such that the starting concentrations were maintained for the duration of the experiment. At 9 dpe, cells were fixed for immunocytochemistry (ICC) and the conditioned medium was collected for analysis of Aβ concentrations by enzyme-linked immunosorbent assay (ELISA). **b** Representative ICC images of neurons at 9 dpe labeled for total tau (red), Aβ (white), and cell nuclei (blue). Scale bars = 20 μm. **c** Percent positive area of Hoechst^+^ cell nuclei at 9 dpe, relative to the DPBS control group. The data represent the mean ± SD of *n* = 6 wells per group. *P < 0.05 by one-way ANOVA. **d** Percent positive area of Aβ^+^ pathology at 9 dpe, relative to the DPBS control group. The data represent the mean ± SD of *n* = 6 wells per group. ***P < 0.001 by one-way ANOVA. **e** Representative ICC images of neurons at 9 dpe to apoE4 and Aβ42 and treated with compounds at 1 μM and labeled for total tau (red), Aβ (white), and cell nuclei (blue). Scale bars = 20 μm. **f** Percent positive area of Hoechst^+^ cell nuclei at 9 dpe, relative to the DMSO control group. The data represent the mean ± SD of *n* = 6 wells for DMSO and *n* = 3 wells per concentration for compounds. *P < 0.05, **P < 0.01, and ***P < 0.001 compared to the DMSO control by one-way ANOVA. **g** Percent positive area of Aβ^+^ pathology at 9 dpe, relative to the DMSO control group. The data represent the mean ± SD of *n* = 6 wells for DMSO and *n* = 3 wells per concentration for compounds. *P < 0.05, **P < 0.01, and ***P < 0.001 compared to the DMSO control by one-way ANOVA

We used an in vitro primary neuron assay to examine the cytotoxicity of the eight small molecule hit compounds (i.e., sulfacetamide, imipramine, EGCG, idarubicin, PD 81723, epirubicin, olanzapine, and indirubin) as well as their efficacy at reducing intracellular and extracellular Aβ neuropathology under conditions more closely resembling the physiological concentrations of Aβ and apoE than were used in the HTS (Fig. 3a). Primary neurons isolated from 5xFAD transgenic mice, which express the human *APP* gene with three familial AD mutations and also express the human *PSEN1* gene with two familial AD mutations [38], were exposed to Aβ42 and/or apoE4, or DPBS as a negative control. Aβ neuropathology developed in the form of intracellular Aβ aggregates, and extracellular Aβ aggregates adhered to cell membranes or culture surfaces, in cultures exposed to Aβ alone or exposed to apoE4+Aβ, whereas no Aβ neuropathology was observed in cultures exposed to apoE4 alone or to DPBS alone (Fig. 3b). It is worth noting that human APP and PSEN1 expression in transgenic mouse neurons begins prior to birth and that the Aβ neuropathology observed is likely comprised of both the Aβ added as a seed and the Aβ produced by the cells. Quantification of cell nuclei revealed that exposure to apoE4+Aβ resulted in a significant reduction in cell viability compared to DPBS, apoE4 alone, or Aβ alone (Fig. 3c). Significantly more Aβ neuropathology was also present in cells exposed to apoE4+Aβ compared to Aβ alone (Fig. 3d), suggesting that apoE4 catalyzes Aβ fibril formation in cell culture medium as it does in acellular assays and that the resulting Aβ fibrils are neurotoxic.

Each of the eight small molecule hit compounds was dosed into the cell culture medium concurrently with exposure to apoE4+Aβ (Fig. 3a). Six of the compounds (i.e., sulfacetamide, imipramine, EGCG, PD 81723, olanzapine, and indirubin) had no discernable effects on cell viability or on neuronal morphology at 9 dpe (Fig. 3e). However, two compounds, idarubicin and epirubicin, caused a significant reduction in cell viability at 0.01 μM (Fig. 3f), which is consistent with their clinical use as topoisomerase II inhibitor chemotherapeutics with known toxicity. These two more toxic compounds may benefit from structural modifications to reduce their side effects while retaining their potent anti-amyloid properties. Sulfacetamide and EGCG produced a slight increase in cell viability at some concentrations, suggesting that they may be neuroprotective. We next examined the effects of the six non-toxic compounds on Aβ neuropathology. At 9 dpe, all six compounds exerted a significant effect on Aβ neuropathology at 100 nM and 1 μM, reducing it by 49–71% compared to the DMSO control, and EGCG, olanzapine, and indirubin also reduced Aβ neuropathology at a concentration of 10 nM (Fig. 3g). Additionally, three compounds (i.e., sulfacetamide, EGCG, and olanzapine) significantly reduced the level of Aβ in the conditioned medium at 9 dpe (Additional file 9), suggesting that they may decrease the cellular production or secretion of Aβ.

### Small molecule compounds reduce pTau neuropathology in primary neurons from TgF344-AD transgenic rats

**Fig. 4:**
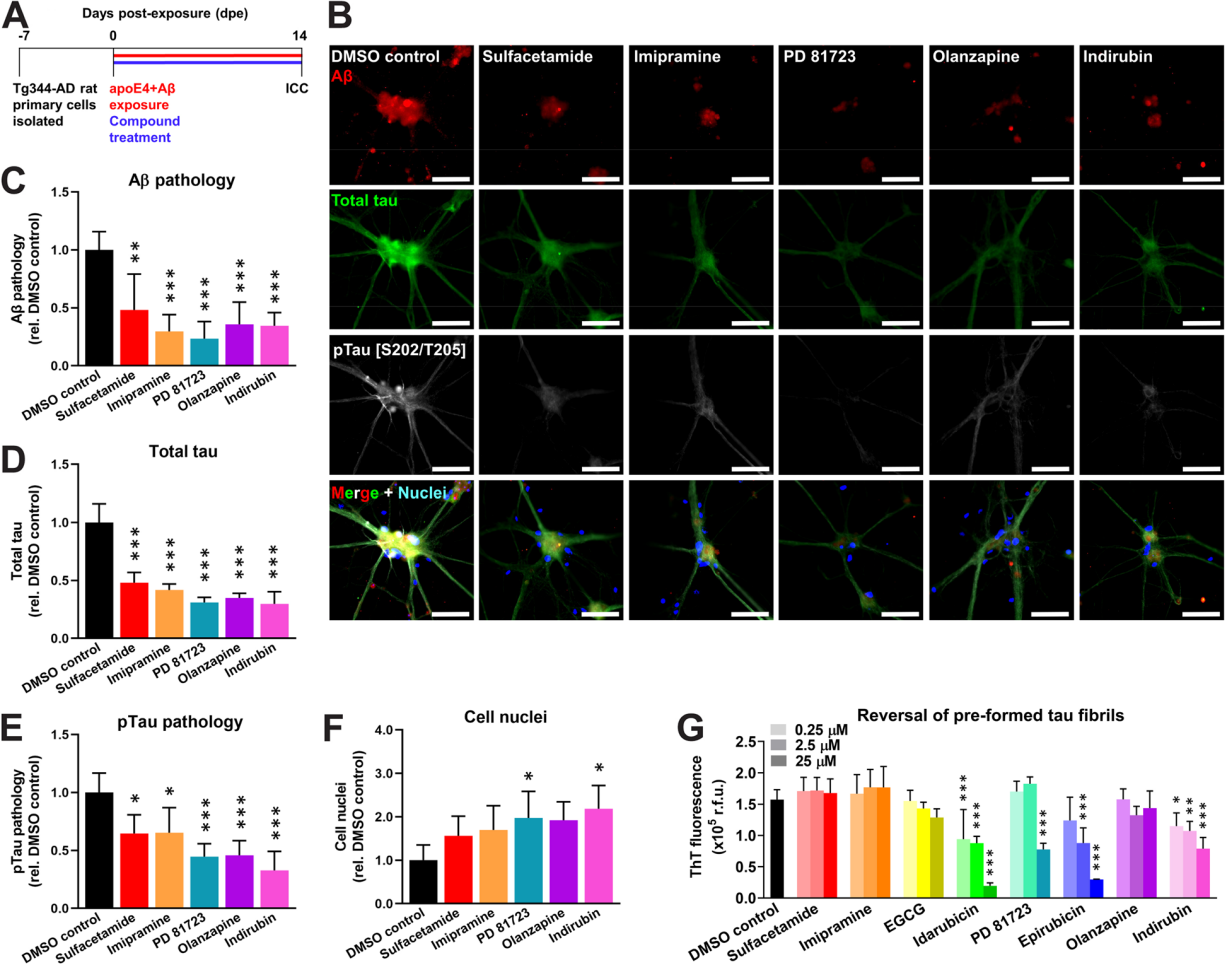
Small molecule compounds inhibit pTau neuropathology in primary neurons from TgF344-AD transgenic rats. **a** Schematic of drug efficacy experiments using primary neurons from the TgF344-AD transgenic rat model of AD. One week after cell isolation from day P1 pups, cells were exposed to 100 nM Aβ42 and 1 nM apoE4 and were treated concurrently with 1 μM compound in a final concentration of 0.5% (v/v) DMSO. The cell medium was changed every 3 days by removing half and replacing it with a fresh medium containing Aβ42, apoE4, and compound such that starting concentrations were maintained for the duration of the experiment. At 14 dpe, cells were fixed for ICC. **b** Representative ICC images of neurons at 14 dpe, treated with compounds at 1 μM, and labeled for Aβ (red), total tau (green), pTau [S202/T205] (white), and cell nuclei (blue). Scale bars = 50 μm. **c** Percent positive area of Aβ^+^ pathology, relative to the DMSO control group. **d** Percent positive area of total tau^+^ fluorescence signal, normalized to the total area of Hoechst^+^ fluorescence signal, and relative to the DMSO control group. **e** Percent positive area of pTau [S202/T205]^+^ pathology, normalized to the total area of Hoechst^+^ fluorescence signal, and relative to the DMSO control group. **f** Total area of Hoechst^+^ cell nuclei at 14 dpe, relative to the DMSO control group. **c**–**f** The data represent the mean ± SD of *n* = 4 wells per group. *P < 0.05, **P < 0.01, and ***P < 0.001 compared to the DMSO control by one-way ANOVA. **g** Eight hit compounds were tested for disaggregation of pre-formed heparin-induced tau fibrils. The experiment was replicated twice, and the results were combined. The data represent the mean ± SD of *n* = 6 wells per group. *P < 0.05, **P < 0.01, and ***P < 0.001 compared to the DMSO control by one-way ANOVA

Aβ induces the phosphorylation and subsequent aggregation of the tau protein into neurofibrillary tangles (NFTs) as a key step in the AD pathogenic process [43]. Because tau aggregation does not occur in 5xFAD mouse neurons, we turned to the TgF344-AD transgenic rat model that expresses human *APP* and *PSEN1* with familial AD mutations and exhibits robust NFT pathology [39]. In a similar experimental paradigm as was used for 5xFAD mice (Fig. 4a), primary neurons from TgF344-AD rats exposed to Aβ and apoE4 formed robust intracellular and extracellular Aβ pathology that was accompanied by pTau neuropathology by 14 dpe, which included intracellular puncta, axonal blebbing, and neuropil thread-like structures (Additional file 10). Following treatment with each of the five novel and non-toxic hit compounds (i.e., sulfacetamide, imipramine, PD 81723, olanzapine, and indirubin), we observed a significant reduction in the amounts of Aβ neuropathology (Fig. 4b, c), total tau (Fig. 4d), and pTau phosphorylated at the S202/T205 epitopes (Fig. 4e) compared to neurons treated with DMSO. Furthermore, treatment with either PD 81723 or indirubin significantly increased neuronal cell survival (Fig. 4f).

To determine whether the hit compounds could act directly on tau, we measured the ability of each compound to disaggregate pre-formed tau fibrils. Idarubicin, PD 81723, epirubicin, and indirubin all significantly reversed tau fibril formation (Fig. 4g). In contrast, sulfacetamide, imipramine, EGCG, and olanzapine had no effect. Taken together, these data indicate that sulfacetamide, imipramine, EGCG, and olanzapine reduce pTau neuropathology via inhibition of Aβ, whereas idarubicin, PD 81723, epirubicin, and indirubin act, at least in part, directly on the tau protein.

### Imipramine and olanzapine use correlates with improved clinical outcomes in human AD patients

We next asked whether any of our identified hit compounds were currently being prescribed for other indications and whether their use was associated with any changes in cognition or risk for developing AD. We acquired longitudinal data from the National Alzheimer’s Coordinating Center (NACC) on 42,661 subjects who were seen at 39 different ADRCs in the USA since 2005 [40]. We searched the prescription drug histories of the subjects in the NACC dataset and found that 40 subjects had taken imipramine, an antidepressant, and that 94 subjects had taken olanzapine, an antipsychotic. We then identified “control” subjects who had been prescribed any antidepressant (*n* = 6233 subjects) or any antipsychotic (*n* = 798 subjects) medication *other* than imipramine or olanzapine, which are listed in Additional file 11. We first evaluated changes in cognition in all of the subjects over time as measured by the MMSE. Controlling for age and sex, we found that the subjects who took imipramine had a significantly greater change (i.e., improvement) in MMSE score over time compared to subjects who took any other antidepressant medication (*P* = 0.0490) (Table 2). Likewise, subjects who took olanzapine had a significantly greater change (i.e., improvement) in MMSE score over time compared to subjects who took any other antipsychotic medication (*P* = 0.0310) (Table 2). Notably, our results show that imipramine use corresponded to an estimated increased score of 0.4186 points (out of 30) per year and that olanzapine use corresponded to an estimated increased score of 0.4937 points per year, relative to their respective control groups (Table 2). Because we identified imipramine and olanzapine as specific inhibitors of the apoE4-Aβ interaction, we also determined whether *APOE* genotype might influence their effects on cognition. When the subjects who took imipramine were segregated into *APOE4* carriers and *APOE4* non-carriers, both groups showed improvement on imipramine by estimate compared to control, and the estimate for *APOE4* carriers was larger, but none of the contrasts were statistically significant (Table 2). Subjects carrying at least one *APOE4* allele who took olanzapine had a significantly greater change (i.e., improvement) in MMSE score over time (*P* = 0.0235), whereas subjects carrying no *APOE4* allele who took olanzapine showed improved cognition by a lower estimate, and it was not statistically significant (Table 2). Similar trends were also observed when the baseline MMSE score was included as a covariate (Additional file 12).

**Table 2:** Retrospective analysis of NACC dataset for cognition and clinical diagnosis reversion or conversion

	Imipramine vs. other antidepressants			Olanzapine vs. other antipsychotics		
	***N*** subjects imipramine; other anti-depressants	Estimate (95% C.I.)	***P***-val	***N*** subjects olanzapine; other anti-psychotics	Estimate (95% C.I.)	***P***-val
**Cognitive exam, ΔMMSE score/year**						
All subjects	40; 6,233	0.4186 (0.0017, 0.8355)	0.0490	94; 798	0.4937 (0.0451, 0.9423)	0.0310
*APOE4* carriers	9; 2,748	0.6017 (-0.3156, 1.5190)	0.1985	51; 354	0.7781 (0.1051, 1.4512)	0.0235
*APOE4* non-carriers	31; 3,485	0.1303 (-0.3812, 0.6419)	0.6175	43; 444	0.3755 (-0.3011, 1.0520)	0.2766
**Clinical diagnosis reversion, hazard ratio**^a^						
All subjects	22, 5,476	1.4487 (1.2280, 1.7092)	<0.0001	74; 679	1.7254 (0.7131, 4.1746)	0.2263
*APOE4* carriers	7; 2,635	0.8667 (0.4757, 1.5790)	0.6401	41; 308	7.0936 (1.0589, 47.5200)	0.0444
*APOE4* non-carriers	15; 2,841	1.5313 (1.3976, 1.6778)	<0.0001	33; 371	1.7422 (0.2738, 11.0870)	0.9983
**Clinical diagnosis conversion, hazard ratio**^b^						
All subjects	23; 3,987	0.9352 (0.7905, 1.1064)	0.9352	24; 191	1.3000 (0.8178, 2.0666)	0.2672
*APOE4* carriers	7; 1,482	0.5286 (0.2585, 1.0808)	0.0806	11; 57	1.9395 (0.9464, 3.9748)	0.0704
*APOE4* non-carriers	16; 2,505	1.1177 (0.9240, 1.3521)	0.2519	13; 134	0.7770 (0.3500, 1.7246)	0.5350
*APOE4* carriers vs. non-carriers	23; 3,987	0.4729 (0.2256, 0.9915)	0.0474	24; 191	2.4963 (0.8532, 7.3033)	0.0949

Cognitive exam (MMSE) scores and clinical diagnosis reversion or conversion were compared between imipramine or olanzapine and control groups using regression modeling and Cox proportional hazard ratios, respectively. Summary statistics for all analyses are provided in Additional file 14. ^a^Only subjects who reported use of a medication prior to reversion to a better clinical diagnosis were included. ^b^Only subjects who reported use of a medication prior to conversion to a worse clinical diagnosis were included

Because one aim of these retrospective analyses is to inform future prospective clinical trials, we used the results of our models in power analyses to estimate the number of AD subjects that would be necessary to observe a similar change in MMSE score over 1 year of dosing with imipramine or olanzapine relative to placebo control groups with 80% power. The results show that 359 AD subjects per group would be necessary in a randomized placebo-controlled clinical trial for imipramine and that 380 AD subjects per group would be necessary in a clinical trial for olanzapine (Additional file 13). Because *APOE4* carriers had greater estimates of change in MMSE score over time than did all subjects (Table 2), our power analyses show that, if only *APOE4* carriers were enrolled, 168 AD subjects per group or 147 AD subjects per group would be necessary for clinical trials of imipramine or olanzapine, respectively.

**Fig. 5:**
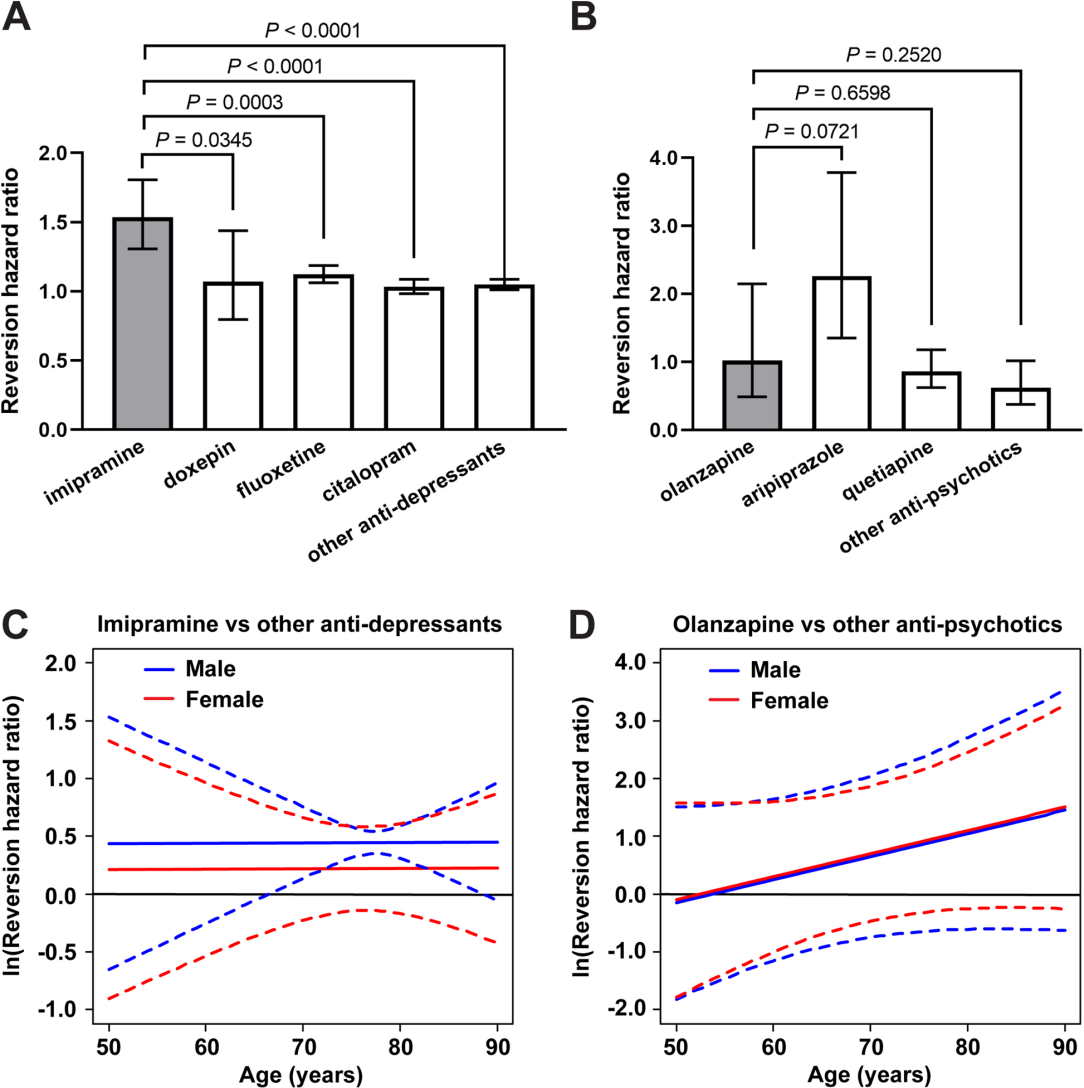
Mixed medication and interaction models evaluating clinical diagnosis reversion. **a** The hazard ratio of clinical diagnosis reversion toward normal was plotted comparing the cumulative drug exposure of imipramine, doxepin, fluoxetine, citalopram, or all other antidepressants to being off the medication, in the same subjects. The data indicate the hazard ratio ± 95% CI. Imipramine was compared to each other group and all *P* values are shown. **b** The hazard ratio of clinical diagnosis reversion toward normal was plotted comparing the effect of being on olanzapine, aripiprazole, quetiapine, or all other antipsychotics to being off the medication, in the same subjects. The data indicate the hazard ratio ± 95% CI. Olanzapine was compared to each other group and all *P* values are shown. **c** Imipramine was compared to other antidepressant medications for the potential effect of cumulative drug exposure on the hazard ratio of clinical diagnosis reversion toward normal, with age and sex considered as interaction variables. The data represent the natural log of the HR (solid lines) and 95% CI (dotted lines). Statistical significance is reached when the 95% CI does not include zero, which occurs from 66.5 to 88.5 years of age in males. **d** Olanzapine was compared to other antipsychotic medications for the potential effect of being on the medication on the hazard ratio of clinical diagnosis reversion, with age and sex considered as interaction variables. The data represent the natural log of the HR (solid lines) and 95% CI (dotted lines)

We next determined whether subjects received an improved clinical diagnosis from their physician after taking imipramine or olanzapine. We used Cox proportional hazards models to evaluate the incidence of a subject reverting from a clinical diagnosis of AD to MCI or reverting from a diagnosis of MCI to NC. Controlling for age and sex, we found that, compared to subjects who took any other antidepressant medication, subjects who took imipramine had an increased incidence of reversion to a better clinical diagnosis by an estimated 44.87% for each additional year of exposure (*P* < 0.0001) (Table 2). *APOE4* carriers who took imipramine also had a significantly decreased incidence of conversion to a worse clinical diagnosis (from NC to MCI or from MCI to AD) compared to *APOE4* non-carriers (*P* = 0.0474) (Table 2). Given that other antidepressants have been previously proposed as AD therapeutics, particularly selective serotonin reuptake inhibitors (SSRIs) [44, 45], we also directly compared imipramine to several other common antidepressants. The incidence of reversion to a better clinical diagnosis was significantly higher for imipramine compared to two common SSRIs, fluoxetine and citalopram, and compared to doxepin, a tricyclic antidepressant with similar pharmacological properties to those of imipramine (Fig. 5a). The incidence of reversion to a better clinical diagnosis when taking olanzapine was an estimated 72.54% greater compared to control antipsychotics, although this result was not statistically significant (Table 2). Among *APOE4* carriers, being on olanzapine increased the incidence of reversion to a better clinical diagnosis by an estimated factor of 7.0936 compared to control (*P* = 0.0444). The incidence of reversion to a better clinical diagnosis while taking olanzapine was not significantly different from the common antipsychotics aripiprazole and quetiapine (Fig. 5b). Interestingly, aripiprazole, which showed the greatest trend toward increased incidence of clinical diagnosis reversion, was a hit in our exploratory drug screen (Additional file 8), although it did not produce a dose-dependent response in our HTS assay (Fig. 2a) and we have not pursued it further.

Finally, we evaluated the relationship between sex and age and the potential effects of imipramine or olanzapine on clinical diagnosis compared to controls. We found that cumulative imipramine exposure significantly increased the incidence of reversion to a better clinical diagnosis for men between the ages of 66.5 and 88.5 years, although the effect was not statistically significant in women (Fig. 5c). Olanzapine showed a trend toward greater benefit for subjects of older age, although the result was not statistically significant (Fig. 5d). Taken together, these results indicate that, compared to other antidepressant and antipsychotic medications, the ability of imipramine and olanzapine to specifically inhibit apoE-catalyzed Aβ fibrillization predicts their specific ability to improve cognition and reverse clinical diagnosis toward normal.

## Discussion

Since the development of ThT-based amyloid fibrillization assays in the 1980s, a wide range of concentrations and assay conditions have been evaluated with no clear consensus [46]. Therefore, our first objective was to determine the optimal conditions of a ThT-based assay for studying the effects of apoE on Aβ fibril formation. We employed DOE, a statistical method for process optimization that allows experimentation on numerous variables at the same time, each at a wide range of values. In contrast to traditional “one variable at a time” methods, DOE is highly efficient and also identifies relevant interactions between variables. As an example of its efficiency, in our first response surface experiment, we evaluated five different Aβ, apoE4, and ThT concentrations each using 32 combinations, rather than testing all 5 × 5 × 5 = 125 possible combinations. In several experiments, each building on the previous one, we ultimately identified 20.9 μM Aβ42, 0.75 nM apoE4, and 14.8 μM ThT as the optimal concentrations that maximize Aβ fibril formation in DPBS at 37°C.

We identified an optimal concentration of 0.75 nM apoE4, which was surprising, given that a much higher concentration of apoE4 was used in the first studies demonstrating that apoE4 accelerates Aβ fibril formation, although other concentrations were not tested [6, 7]. The physiological concentrations of apoE in human plasma and cerebrospinal fluid are approximately 4 μM and 100 nM, respectively [47, 48]. However, it is important to consider that apoE and Aβ are most likely to interact in the brain interstitial fluid, especially around synapses where Aβ is produced and exerts its neurotoxic effects [49]. Thus, the most relevant benchmark may be the apoE concentration in brain interstitial fluid, which has been shown to be 0.30 nM for wild-type mice and 0.37 nM for human *APOE4* knock-in mice, as measured by in vivo microdialysis [50]. When 5xFAD mice were crossed with the same *APOE4* knock-in mice, they showed significantly accelerated plaque deposition [51], suggesting that apoE concentrations similar to those that were tested here are sufficient to catalyze Aβ fibrillization in situ. With respect to the low apoE4:Aβ ratio used in our experiments, it should be noted that apoE has two binding sites for Aβ [52] and that Aβ42 often exists in a polymeric β-sheet structure in the AD brain, for which apoE has greater affinity [53]. Based on the traditional definition of a catalyst, we have also hypothesized that apoE may not be consumed in the catalytic reaction, but may instead be released from Aβ and thereby catalyze the formation of multiple fibrils [54], although this has yet to be demonstrated. In this case, very low concentrations of apoE4 may exert a significant amyloidogenic effect, underscoring the importance of inhibiting its interaction with Aβ. Our results also indicate that the catalytic effect of apoE is highly dependent on the apoE to Aβ molar ratio, which may explain, in part, conflicting reports on its amyloidogenic effects in vitro [55].

Our HTS assay identified eight compounds with potent activity against Aβ aggregation or against the catalytic effect of apoE4 on Aβ fibrillization. Interestingly, our reversal studies then showed that five of those compounds — EGCG, idarubicin, PD 81723, epirubicin, and indirubin — disaggregated pre-formed fibrils of Aβ (Fig. 2b–e), which suggests that these molecules may be pursued as interventional treatments for patients with pre-existing AD neuropathology. On the other hand, we found that sulfacetamide, imipramine, and olanzapine did not block or reverse Aβ fibrillization independent of apoE4, suggesting that they are specific inhibitors of the apoE4-Aβ interaction and warrant further development for preventing AD, particularly in *APOE4* carriers. We then tested all eight hit compounds in primary neurons from 5xFAD mice that overproduce human Aβ leading to both intraneuronal and extracellular Aβ neuropathology [38], which was accelerated by the addition of human apoE4 and Aβ42 to the culture medium as seeds. ApoE is capable of penetrating the cell membrane and enhancing neuronal Aβ uptake [56, 57] and may have thereby contributed to the intraneuronal aggregation of Aβ. Human apoE4 itself has been reported to be toxic to neurons in culture and in mice [56], but we did not observe this effect at the very low concentration we used. Therefore, we believe that the effects of apoE4 in our neuronal assays were predominantly via catalysis of Aβ fibril formation. Despite using isolation and culture methods that favored neurons, sparse astrocytes, which secrete apoE, are often present in neuronal cultures, and neurons also secrete apoE under stressed conditions [58]. Therefore, we cannot rule out a potential contribution of mouse- or rat-derived apoE to the Aβ and pTau neuropathologies observed in our cellular assays. We found that all six non-toxic hit compounds, including sulfacetamide, imipramine, EGCG, PD 81723, olanzapine, and indirubin, reduced Aβ pathology in 5xFAD mouse neurons (Fig. 3g), either by inhibiting the effect of apoE4 or by preventing/reversing Aβ aggregation, and we then confirmed this effect in a second model using Tg344 rat primary neurons (Fig. 4c). Importantly, we also showed that the compounds reduced the subsequent intraneuronal accumulation of pTau protein (Fig. 4d,e), which is directly linked to neurodegeneration and cognitive decline [59, 60]. The effects of PD 81723 and indirubin on pTau pathology may have been, in part, via direct action on tau oligomers/fibrils. However, sulfacetamide, imipramine, and olanzapine showed no direct reversal effects on tau fibrils (Fig. 4g), indicating that they most likely reduce pTau pathology and subsequent neurodegeneration indirectly via inhibition of the effect of apoE4 on Aβ.

Imipramine is a tricyclic antidepressant that blocks norepinephrine and serotonin reuptake. Given the frequent use of antidepressants by AD patients, imipramine has also been evaluated in in vitro and in vivo models of AD, where it has been found to reduce Aβ accumulation and cognitive deficits [61–63]. Olanzapine is an antipsychotic drug that has been evaluated for acute treatment of behavioral and psychological symptoms of AD [64]. Olanzapine has not been tested clinically as a disease-modifying therapy for AD, but it has been shown to have neuroprotective effects against Aβ-induced oxidative stress and apoptosis [65, 66]. Indirubin, a natural compound, was found to both prevent and to reverse Aβ fibrillization in our studies. Indirubin is best known for being a potent inhibitor of cyclin-dependent kinases (CDKs) and glycogen synthase kinase-3β (GSK-3β), both of which phosphorylate tau. Therefore, indirubin may have multi-functional therapeutic benefits for AD. Indeed, indirubin has been reported to reduce amyloid and tau pathology, attenuate neuroinflammation, and improve spatial memory deficits in AD mouse models [67]. Imipramine, olanzapine, and indirubin demonstrated efficacy in our cellular Aβ assay at nanomolar concentrations (Fig. 3g). These compounds are exceptionally promising because they may accommodate peripheral dosing, for which central nervous system (CNS) bioavailability is very low, even for BBB-permeable drugs. Maintaining a therapeutic drug concentration in the brain is crucial because inhibiting peripheral apoE or increasing its levels by parabiosis has been shown to have no effect on Aβ deposition in the brain [68, 69]. We also identified sulfacetamide, an antibiotic, and PD 81723, an allosteric enhancer of brain adenosine A1 receptors, as novel therapeutic candidates that have not been evaluated previously for AD.

Depression and psychosis are well-known co-morbidities of AD and other dementias. As such, a significant proportion of NACC participants reported the use of antidepressant and/or antipsychotic medications, providing large control populations with similar clinical presentations that enabled us to evaluate the potential effects of imipramine and olanzapine. Our analyses show that, compared to the control populations, subjects taking imipramine or olanzapine had improved cognition and diagnoses, which are direct clinical measures of disease severity. Notably, in our drug screen, we found that imipramine and olanzapine strongly inhibited the apoE4-catalyzed fibrillization of Aβ, whereas none of the other antidepressants or antipsychotics in the NCC library had any such activity. In line with our identified mechanism of action, these apoE4 inhibitors also demonstrated a preferential benefit for *APOE4* carriers over non-carriers, with those taking olanzapine having a greater change (i.e., improvement) in MMSE score and those taking imipramine having reduced incidence of conversion to a worse clinical diagnosis (Table 2). Given that the levels of neurotoxic Aβ oligomers have been shown to be increased in the brains of *APOE4* carriers [19, 20], it is particularly important that the drugs appear to be effective in this population. Furthermore, cumulative imipramine exposure was associated with a significantly greater incidence of reversion to a better clinical diagnosis compared to fluoxetine and citalopram (Fig. 5a), two SSRIs proposed to reduce Aβ production via increased serotonin signaling that have been evaluated in humans [70]. Taken together, these results provide strong evidence of the potential clinical benefits of imipramine and olanzapine use in human subjects and support further development and evaluation of these and our other hit compounds as disease-modifying treatments for AD.

The clinical diagnoses recorded in the NACC database were frequently made in consensus conferences, wherein at least one physician and one neuropsychologist evaluated a subject’s MMSE score, neuropsychological exam, and full clinical history, among other information [40]. The fact that we found both imipramine and olanzapine use to be associated with improvements in clinical diagnosis, for which MMSE (a memory-focused exam) was weighed only in part, suggests that these drugs may have had additional functional benefits not identified here, but which were taken into account in the consensus conferences. It is also possible that beneficial neuropsychological effects of imipramine and olanzapine, via their primary mechanisms of action, could have contributed to the improvements observed in our study. However, imipramine and olanzapine have not been found to be particularly effective for treating depression or psychosis in AD patients [64, 71]. Both drugs have known interactions and side effects and are prescribed cautiously in elderly patients [72], which is likely a reflection of the dosages necessary to achieve their antidepressant or antipsychotic effects and may be alleviated or avoided in clinical trials for AD. Our power analyses indicate that 359 and 380 AD subjects would be appropriate for 1-year-long clinical trials of imipramine and olanzapine, respectively; however, if only *APOE4* carriers were recruited, the sample sizes could be reduced to 168 and 147 AD subjects for imipramine and olanzapine, respectively (Additional file 13). By comparison, the recent ENGAGE (NCT02477800) and EMERGE (NCT02484547) trials of aducanumab for AD enrolled more than 1000 subjects per treatment group. Our results, taken together with in vivo data showing efficacy of imipramine in rodent models of AD [61, 62], suggest that prospective clinical studies of imipramine and olanzapine for the prevention/reversal of AD are warranted and could be accomplished relatively quickly and inexpensively with low risk of adverse events.

Novel CNS drug development has historically been a 10- to 17-year-long process with less than a 10% chance of success and a cost of approximately $1.8 billion per drug [73]. For drugs targeting AD, novel drug development has been especially challenging due to the slow progression of disease requiring lengthy clinical trials with a large number of participants and due to the lack of robust and predictive biomarkers [74]. Although a number of drugs targeting Aβ are currently being tested, there has been a very high failure rate of ~99.6% for AD therapeutics in clinical trials, and there are currently no approved disease-modifying treatments for AD [74]. Drug repurposing, using known drugs for novel indications, has several unique advantages for AD. There is existing knowledge from prior clinical trials on the pharmacological effects, pharmacokinetics, toxicology, and side effects in humans. Therefore, drugs with good safety profiles can be prioritized, expediting the early phases of clinical testing and reducing the failure rate. Development costs are significantly less for repurposed drugs, increasing the chances that a company will be willing to invest to bring a drug to market. For these reasons, a repurposed drug in phase II trials has greater than twice the likelihood of making it to market than a novel drug [75]. Indeed, one of the most widely prescribed medications to reduce some symptoms of dementia is memantine, which was originally developed as an antiviral drug and was then serendipitously found to have anti-glutamatergic activity and was repurposed for AD [76]. Other high-content phenotypic screens have been developed in recent years aimed at drug repurposing for AD [77]; however, none has focused on inhibition of apoE as a key driver of disease. For the reasons highlighted above, the methods used here to identify drug candidates with some safety/dosing information available, good BBB permeability, and strong preclinical efficacy position them well to reach the clinic as disease-modifying therapeutics for AD.

### Limitations

Despite careful planning, our study has several limitations that should be considered. First, the exploratory drug screen was not replicated twice due to time and cost considerations. However, all hits were subsequently tested for dose-response in the kinetic HTS assay which identified the eight top hit compounds. Similarly, the experiments with 5xFAD mouse neurons were not replicated twice, yet the non-toxic hit compounds were subsequently tested in Tg344-AD rat cultured neurons which yielded the same results. Second, our determination as to whether each hit compound acts on Aβ or on apoE was based on their inhibitory effects in the presence or absence of apoE4, rather than on binding studies. Such studies are planned but are also complicated by the dynamic nature of Aβ polymerization, making it difficult to delineate between Aβ monomers, oligomers, fibrils, and potentially unique apoE-catalyzed Aβ structures. Furthermore, our in vitro experiments utilized recombinant apoE proteins, whereas the degree of apoE lipidation is known to affect its role in Aβ aggregation [78] and should be studied in future experiments. Third, in our retrospective analyses of the NACC clinical dataset, our sample sizes for the imipramine and olanzapine groups were small relative to a typical prospective clinical trial. The NACC dataset is the largest available clinical dataset of AD patients, and we included every eligible subject who reported imipramine or olanzapine use and who had at least two clinical records which allowed us to evaluate change over time. Despite the relatively small sample sizes, the fact that some models had *P* values less than 0.0001 (e.g., imipramine, reversion) or very large effect sizes (e.g., olanzapine, reversion, *APOE4* carriers) gives confidence in our overall conclusions. Importantly, larger sample sizes would increase the power and precision of the effect size estimates, but would not drastically change the effect sizes or *P* values. We attempted to replicate our findings in other clinical datasets containing medication records (e.g., the Alzheimer’s Disease Neuroimaging Initiative); however, they contained many fewer subjects overall and thus very few subjects who reported taking imipramine or olanzapine. Fourth, it is possible that a clinician’s prescription of a certain antidepressant or antipsychotic may have been determined by patient co-morbidities (e.g., diabetes, hypertension, etc.) that could have had independent effects on cognition. Alternatively, imipramine or olanzapine may have had effects on cognition by apoE-independent mechanisms that have yet to be determined. However, our review of the literature and discussions with clinicians have revealed no such preference for the prescription of imipramine or olanzapine or regarding their ability to have the observed effects on human cognition and AD diagnosis. We have yet to evaluate imipramine in rodent AD models. Although previous in vivo studies using AD rodent models [61, 62] and the clinical evidence provided herein are promising, prospective randomized controlled trials will be necessary to determine the efficacy and dosing of imipramine and the other hit compounds as AD-modifying therapies (rather than their original indications) in a more controlled population.

## Conclusions

These biochemical, cellular, and clinical results strongly support the concept that apoE serves as a catalyst for fibrillization of Aβ into neurotoxic oligo/polymers and that further studies on this approach to the development of AD therapeutics are warranted. Furthermore, apoE has been implicated in a number of Aβ-independent pathogenic mechanisms that cause Parkinson’s disease, primary tauopathies, and amyotrophic lateral sclerosis, among other disorders [79]. Thus, the apoE-centric screening methods and drug candidates we report here may also prove valuable for addressing other human neurodegenerative diseases.

## Supplementary Information

**Additional file 1.** Half-fraction factorial design. Three reactant concentrations, Aβ, apoE4, and ThT, were varied in a half-fraction factorial design for a total of 2^3^/2 = 4 experimental conditions and one center point. Three technical replicates (wells) were tested per experimental condition. Experimental conditions and data are provided in Additional file 6. a, Pareto chart of the standardized effect for each reactant on the integrated AUC of ThT intensity. The critical effect size for statistical significance (α = 0.05) is also shown at an effect size of 2.23 (red line). Aβ concentration had a large effect while the effects of apoE4 and ThT concentrations were insignificant. The interaction effects are confounded with the main effects and are therefore not shown. b, Main effects plot showing the size and direction of each effect on the AUC of ThT intensity. As Aβ concentration increased from 1 μM to 20 μM the AUC of ThT intensity increased from 0 to approximately 1.6 x 10^6^ a.u., while apoE4 and ThT concentrations had no significant effects.

**Additional file 2.** Central composite response surface design #1. The concentrations of three reactants, Aβ, apoE4, and ThT, were varied in a central composite design using 2^3^ = 8 corner points, 2*3 = 6 axial points, and one center point. An optimized design space was determined based on the results of the previous factorial experiment. Two replicates (wells) were tested per experimental condition and four replicates of the center point. Experimental conditions and data are provided in Additional file 6. a,b, Pareto charts showing the standardized effect for the main (A, B, C), quadratic (AA, BB, CC), and interaction effects (AB, BC, AC) on the AUC and the fold-change of ThT intensity, respectively. The critical effect size for statistical significance (α = 0.05) is also shown at an effect size of 2.074 (red line). All three variables had large main and quadratic effects on the AUC of ThT intensity, while all interaction effects were negligible. ThT and Aβ concentrations had large main and quadratic effects on the fold-change in ThT intensity, while the effects of apoE concentration and all interaction effects were much smaller. c,b, Main effects plots showing the combination of main and quadratic effects of each reactant on the AUC and the fold-change of ThT intensity, respectively. High Aβ concentration, low apoE4 concentration, and an intermediate ThT concentration maximized both the AUC, and the fold-change, of ThT intensity. e,f, Interaction plots showing the interaction effect of each reactant pair on the AUC and the fold-change of ThT intensity, respectively. No significant interactions were observed, which is evidenced by the similar shapes of the response curves for all reactant concentrations in each interaction plot.

**Additional file 3.** Effects of apoE4 concentration on Aβ42 fibrillization. Concentrations of Aβ42, apoE4, and ThT were varied in a response surface design. The fibrillization assay was run in a 384-well plate and was analyzed for ThT fluorescence over a 24 h period. Several groups were plotted to demonstrate the effects of the different concentrations of apoE4 on ThT fluorescence over time. The complete results are provided in Additional file 6. The data represent the mean of n = 3−4 wells per group.

**Additional file 4.** Central composite response surface design #2. The concentrations of three reactants, Aβ, apoE4, and ThT, were varied in a central composite design using 2^3^ = 8 corner points, 2*3 = 6 axial points, and one center point. An optimized design space was determined based on the results of the previous response surface experiment. Three replicates (wells) were tested per experimental condition and six replicates of the center point, and the entire experiment was repeated in two independent experiments (blocks). Experimental conditions and data are provided in Additional file 6. a,b, Pareto charts showing the standardized effect for the main (A, B, C), quadratic (AA, BB, CC), and interaction effects (AB, BC, AC) on the AUC and the fold-change of ThT intensity, respectively. The critical effect size for statistical significance (α = 0.05) is also shown at an effect size of 2.447 (red line). Aβ and ThT had large main and quadratic effects on the AUC of ThT intensity, while the effect of apoE was smaller. Aβ had the largest main and quadratic effects on the fold-change in ThT intensity, while the effects of apoE and ThT were smaller. c,d, Main effects plots showing the combination of main and quadratic effects of each reactant on the AUC and the fold-change of ThT intensity, respectively. Intermediate concentrations of Aβ, apoE4, and ThT maximized both the AUC and the fold-change of ThT intensity. e,f, Interaction plots showing the interaction effect of each reactant pair on the AUC and the fold-change of ThT intensity, respectively. An interaction between Aβ and ThT concentrations (AC) was observed to have a moderate effect on both the AUC and the fold-change of ThT intensity, which is evidenced by the response curves for different reactant concentrations crossing one another. This moderate effect caused both responses to peak at lower Aβ concentrations when the ThT concentration was 20 μM compared to 10.5 μM. However, the interaction effect did not change the conclusions about the dominant main and quadratic effects of Aβ and ThT seen in the main effects plot.

**Additional file 5.** Effect of DMSO in the optimized apoE4-Aβ fibrillization assay. The effects of DMSO at 0, 1, 5, and 10% (v/v) on apoE4-catalyzed Aβ42 fibrillization were evaluated. The data represent the mean ± SD of n = 8 wells per group.

**Additional file 6.** Design of experiments (DOE) assay optimization data. Experimental conditions and raw data for (A) Half-fraction factorial experiment, (B) Response surface experiment #1, and (C) Response surface experiment #2. The RunOrder column indicates randomized order in which the different experimental conditions were prepared in the wells of a plate. The CenterPt column indicates whether the experimental condition is a center point (0) or not (1). The PtType column indicates whether the experimental condition is a corner or axial point (-1 or 1), or a center point (0). The Blocks column indicates whether the experimental condition was included in a single plate run on one day (1) or was included in a second plate repeating the entire experiment on a different day (2). The AUC ThT intensity column indicates the integrated area under the curve of ThT fluorescence intensity measured by the plate reader in arbitrary units (a.u.) over the entire experiment duration. The Fold-change ThT intensity column indicates the fold-change in ThT fluorescence intensity from the beginning to the end of the experiment.

**Additional file 7 **Exploratory drug screen. a, The apoE4/Aβ42 fibrillization assay was performed in an endpoint fashion in the exploratory screen. To set up the fibrillization assay, Aβ42 (2 μM) and apoE4 (20 nM) were combined in water in a 96-well plate and incubated for 15 min. ThT and glycine were added and incubated for 10 min, and then fluorescence was measured at λ_ex_ = 440 nm, λ_em_ = 490 nm. (A) Under these conditions, Aβ42+apoE4 resulted in significantly greater ThT fluorescence than Aβ42, apoE4, or ThT alone. The data represent the mean ± SD of n = 3 wells per group. Statistical significance is indicated as **P < 0.01, ***P < 0.001 by one-way ANOVA. b, In the exploratory screen, compounds (2 μM), or DMSO as the control, were initially incubated with Aβ42 and apoE4, and the ThT intensity for each compound was normalized to the control group on the same plate. A total of 595 compounds from the NCC library were evaluated in the exploratory screen, with 134 being identified as hit compounds (red dots) and 461 being identified as non-hit compounds (blue dots). Each data point represents a single compound tested in n = 3 wells and averaged, and the black lines indicate the mean ± SD for each group.

**Additional file 8.** Exploratory drug screen results. (A) Detailed information about the 134 hit compounds identified in the exploratory screen. (B) BBB permeability for the 134 hit compounds identified in the exploratory screen, as determined by a literature search. Positive BBB qualities were found for 87 compounds (labeled in green), negative BBB qualities were found for 41 compounds (labeled in red), and no information was found for 6 compounds (labeled in yellow).

**Additional file 9 **Aβ levels in conditioned medium of 5xFAD mouse neurons. Aβ42 concentrations were measured in the conditioned medium of 5xFAD mouse neurons at 9 dpe to each hit compound by enzyme-linked immunosorbent assay (ELISA). The data represent the mean ± SD of n = 6 wells for the DMSO control and n = 3 wells per concentration for each compound. Statistical significance is indicated as *P < 0.05, **P < 0.01, and ***P < 0.001 compared to the DMSO control by one-way ANOVA.

**Additional file 10.** pTau neuropathological features observed in TgF344-AD primary rat neurons. a−c, Representative ICC images of neurons at 14 dpe to apoE4 and Aβ42, treated with DMSO only as a control, and labeled for Aβ (red), total tau (green), pTau [S202/T205] (white), and cell nuclei (blue). Characteristic pTau neuropathological features were observed including (a) intracellular and extracellular puncta, (b) axonal blebbing, and (c) neuropil thread-like structures. Arrowheads indicate respective pTau neuropathological features. Scale bars = 50 μm.

**Additional file 11.** Other antidepressant and antipsychotic medications used to define the control subject groups. Medications listed under the “NACCADEP” and “NACCAPSY” variables in the NACC dataset.

**Additional file 12.** Retrospective analysis of NACC dataset for cognition including baseline MMSE covariates. The cumulative exposure of imipramine and other antidepressants were compared, and the on/off status of olanzapine and other antipsychotics were compared, using regression modeling for statistical comparisons of cognitive exam and including the baseline MMSE score as a covariate.

**Additional file 13.** Power analyses for a hypothetical one year-long clinical trial of imipramine or olanzapine in AD subjects. Outputs from the Cox regression models of change in MMSE score over time, including the effect size and the standard deviation, were used in power analyses to estimate the sample sizes necessary to detect a similar difference between imipramine or olanzapine and control groups with an α (type 1 error level) of 0.05 and a β (type 2 error level) of 0.2. The sample sizes indicate the number of AD subjects that would be dosed with imipramine or olanzapine for one year with an equal number of control AD subjects receiving placebo.

**Additional file 14.** Summary statistics of NACC data analyses. (A, B) Subject information for MMSE models comparing (A) imipramine and other antidepressants or (B) olanzapine and other antipsychotics, including age, sex, baseline MMSE score, and drug exposure time. (C, D) Subject information for clinical diagnosis reversion models comparing (C) imipramine and other antidepressants or (D) olanzapine and other antipsychotics, including age, sex, baseline MMSE score, drug exposure time, number of subjects with reversions, and number of reversions per subject. (E, F) Subject information for clinical diagnosis conversion models comparing (E) imipramine and other antidepressants or (F) olanzapine and other antipsychotics, including age, sex, baseline MMSE score, drug exposure time, number of subjects with conversions, and number of conversions per subject. (G, H) Subject information for multiple medications models comparing (G) imipramine, doxepin, fluoxetine, citalopram, and all other antidepressants or (H) olanzapine, aripiprazole, quetiapine, and all other antipsychotics, including age, sex, baseline MMSE score, drug exposure time, number of subjects with reversions, and number of reversions per subject. (I) Complete test statistics and degrees of freedom for all statistical tests.

**Additional file 15.** Custom computer code generated for NACC data analysis. Computer code written in SAS and used to perform all statistical analyses of NACC data is provided in a text file. Computer code written in R and used to generate plots of NACC data is provided as an R file.

## Data Availability

All data generated or analyzed during this study involving the drug screen are included in this published article and its additional files. The NACC data supporting the findings of this study are available by request from www.naccdata.org. All code generated during this study is included in Additional file 15.

## Abbreviations

Aβ: Amyloid-beta
Aβ40: 40 amino acid amyloid-beta peptide
Aβ42: 42 amino acid amyloid-beta peptide
AD: Alzheimer’s disease
ADRCs: Alzheimer’s Disease Research Centers
ANOVA: Analysis of variance
apoA-I: Apolipoprotein A-I
apoE: Apolipoprotein E
APP: Amyloid precursor protein
AUC: Area under the curve
BBB: Blood-brain barrier
BSA: Bovine serum albumin
CNS: Central nervous system
DMSO: Dimethyl sulfoxide
DOE: Design of experiments
DPBS: Dulbecco’s phosphate-buffered saline
dpe: Days post-exposure
EGCG: Epigallocatechin gallate
ELISA: Enzyme-linked immunosorbent assay
FDA: Food and Drug Administration
HTS: High-throughput screening
MCI: Mild cognitive impairment
NACC: National Alzheimer’s Coordinating Center
NaOH: Sodium hydroxide
NC: Normal cognition
NCC: National Institutes of Health Clinical Collection
NFT: Neurofibrillary tangle
NIH: National Institutes of Health
MMSE: Mini-Mental State Exam
PSEN1: Presenilin 1
pTau: Phosphorylated tau
SSRI: Selective serotonin reuptake inhibitor
TEM: Transmission electron microscopy
ThT: Thioflavin T
