# Supplementary material for: Imipramine and olanzapine block apoE4-catalyzed polymerization of Aβ and show evidence of improving Alzheimer’s disease cognition
Source: Alzheimers Res Ther. 2022 Jun 29;14:88. doi: 10.1186/s13195-022-01020-9 (PMC9241285; doi:10.1186/s13195-022-01020-9)
Supplement: Supplementary file 11 — Additional file 11. Other antidepressant and antipsychotic medications used to define the control subject groups. Medications listed under the “NACCADEP” and “NACCAPSY” variables in the NACC dataset. [file 13195_2022_1020_MOESM11_ESM.docx]

| **Other antidepressants** | **Other antipsychotics** |
| --- | --- |
| bupropion  St. Johns wort  5-hydroxytryptophan  vilazodone  fluoxetine  sertraline  paroxetine  fluvoxamine  citalopram  escitalopram  nortriptyline  desipramine  amitriptyline  doxepin  trimipramine  amoxapine  protripyline  clomipramine  isocarboxazid  phenelzine  tranylcypromine  selegiline  trazodone  nefazodone  maprotiline  mirtazapine  venlafaxine  duloxetine  milnacipran  desvenlafaxine  levomilnacipran | haloperidol  lithium  molindone  loxapine  pimozide  amitriptyline-chlordiazepoxide  amitriptyline-perphenazine  fluoxetine-olanzapine  chlorpromazine  fluphenazine  prochlorperazine  promazine  thioridazine  methotrimeprazine  perphenazine  mesoridazine  trifluoperazine  triflupromazine  thiothixene  clozapine  risperidone  quetiapine  ziprasidone  aripiprazole  paliperidone  iloperidone  asenapine  lurasidone |

**Additional file 11. Other antidepressant and antipsychotic medications used to define the control subject groups**. Medications listed under the “NACCADEP” and “NACCAPSY” variables in the NACC dataset.
