# Supplementary material for: Imipramine and olanzapine block apoE4-catalyzed polymerization of Aβ and show evidence of improving Alzheimer’s disease cognition
Source: Alzheimers Res Ther. 2022 Jun 29;14:88. doi: 10.1186/s13195-022-01020-9 (PMC9241285; doi:10.1186/s13195-022-01020-9)
Supplement: Supplementary file 12 — Additional file 12. Retrospective analysis of NACC dataset for cognition including baseline MMSE covariates. The cumulative exposure of imipramine and other antidepressants were compared, and the on/off status of olanzapine and other antipsychotics were compared, using regression modeling for statistical comparisons of cognitive exam and including the baseline MMSE score as a covariate. [file 13195_2022_1020_MOESM12_ESM.docx]

|  | **Imipramine vs. other antidepressants** | | |  | **Olanzapine vs. other antipsychotics** | | |
| --- | --- | --- | --- | --- | --- | --- | --- |
|  | ***N* subjects imipramine; other anti-depressants** | **Estimate**  **(95% C.I.)** | ***P*-val** |  | ***N* subjects olanzapine; other anti-psychotics** | **Estimate**  **(95% C.I.)** | ***P*-val** |
| **Cognitive exam, ΔMMSE score/year** | | | | | | | |
| All subjects | 40; 6,299 | 0.3295  (-0.0792, 0.7381) | 0.1140 |  | 94; 793 | 0.4316  (-0.0146, 0.8778) | 0.0580 |
| *APOE4* carriers | 9; 2,733 | 0.0559  (-0.4456, 0.5575) | 0.8270 |  | 51; 352 | 0.6004  (-0.0683, 1.2692) | 0.0784 |
| *APOE4* non-carriers | 31; 3,467 | 0.5602  (-0.3377, 1.4582) | 0.2214 |  | 43; 441 | 0.3417  (-0.3306, 1.0139) | 0.3191 |

**Additional file 12. Retrospective analysis of NACC dataset for cognition including baseline MMSE covariates.** The cumulative exposure of imipramine and other antidepressants were compared, and the on/off status of olanzapine and other antipsychotics were compared, using regression modeling for statistical comparisons of cognitive exam and including the baseline MMSE score as a covariate.
