# Supplementary material for: Imipramine and olanzapine block apoE4-catalyzed polymerization of Aβ and show evidence of improving Alzheimer’s disease cognition
Source: Alzheimers Res Ther. 2022 Jun 29;14:88. doi: 10.1186/s13195-022-01020-9 (PMC9241285; doi:10.1186/s13195-022-01020-9)
Supplement: Supplementary file 13 — Additional file 13. Power analyses for a hypothetical one year-long clinical trial of imipramine or olanzapine in AD subjects. Outputs from the Cox regression models of change in MMSE score over time, including the effect size and the standard deviation, were used in power analyses to estimate the sample sizes necessary to detect a similar difference between imipramine or olanzapine and control groups with an α (type 1 error level) of 0.05 and a β (type 2 error level) of 0.2. The sample sizes indicate the number of AD subjects that would be dosed with imipramine or olanzapine for one year with an equal number of control AD subjects receiving placebo. [file 13195_2022_1020_MOESM13_ESM.docx]

|  | **Imipramine vs. placebo** | | | | | |  | | **Olanzapine vs. placebo** | | | | | |
| --- | --- | --- | --- | --- | --- | --- | --- | --- | --- | --- | --- | --- | --- | --- |
|  | **Effect size** | **Std dev** | **α** | **β** | **Sample size** |  | | **Effect size** | | **Std dev** | **α** | **β** | **Sample size** |  |
| All subjects | 0.4186 | 2.0013 | 0.05 | 0.2 | 359 |  | | 0.4937 | | 2.4281 | 0.05 | 0.2 | 380 |  |
| *APOE4* carriers | 0.6017 | 1.9584 | 0.05 | 0.2 | 168 |  | | 0.7781 | | 2.3691 | 0.05 | 0.2 | 147 |  |

**Additional file 13. Power analyses for a hypothetical one year-long clinical trial of imipramine or olanzapine in AD subjects.** Outputs from the Cox regression models of change in MMSE score over time, including the effect size and the standard deviation, were used in power analyses to estimate the sample sizes necessary to detect a similar difference between imipramine or olanzapine and control groups with an α (type 1 error level) of 0.05 and a β (type 2 error level) of 0.2. The sample sizes indicate the number of AD subjects that would be dosed with imipramine or olanzapine for one year with an equal number of control AD subjects receiving placebo.
